# THOR’s Hammer: the Antibiotic Koreenceine Drives Gene Expression in a Model Microbial Community

**DOI:** 10.1128/mbio.02486-21

**Published:** 2022-04-18

**Authors:** Amanda Hurley, Marc G. Chevrette, Natalia Rosario-Meléndez, Jo Handelsman

**Affiliations:** a Wisconsin Institute for Discovery and Department of Plant Pathology, University of Wisconsin—Madison, Madison, Wisconsin, USA; b Wisconsin Institute for Discovery and the Microbiology Doctoral Training Program, University of Wisconsin—Madison, Madison, Wisconsin, USA; McMaster University

**Keywords:** antibiotic signaling, microbial communities, rhizosphere microbes, transcriptional regulation

## Abstract

Microbial interactions dictate the structure and function of microbiomes, but the complexity of natural communities can obscure the individual interactions. Model microbial communities constructed with genetically tractable strains known to interact in natural settings can untangle these networks and reveal underpinning mechanisms. Our model system, The Hitchhikers of the Rhizosphere (THOR), is composed of three species—Bacillus cereus, Flavobacterium johnsoniae, and Pseudomonas koreensis—that co-isolate from field-grown soybean roots. Comparative metatranscriptomics on THOR revealed global patterns of interspecies transcriptional regulation. When grown in pairs, each member of THOR exhibits unique signaling behavior. In the community setting, gene expression is dominated by pairwise interactions with Pseudomonas koreensis mediated either directly or indirectly by its production of the antibiotic koreenceine—the apparent “hammer” of THOR. In pairwise interactions, the koreenceine biosynthetic cluster is responsible for 85 and 22% of differentially regulated genes in *F. johnsoniae* and B. cereus, respectively. Although both deletion of the koreenceine locus and reduction of *P. koreensis* inoculum size increase *F. johnsoniae* populations, the transcriptional response of *P. koreensis* is only activated when it is a relative minority member at the beginning of coculture. The largest group of upregulated *P. koreensis* genes in response to *F. johnsoniae* are those without functional annotation, indicating that focusing on genes important for community interactions may offer a path toward functional assignments for unannotated genes. This study illustrates the power of comparative metatranscriptomics of microorganisms encountering increasing microbial complexity for understanding community signal integration, antibiotic responses, and interspecies communication.

## INTRODUCTION

The planet is replete with microbial communities, or microbiomes, that drive the functions of their habitats. Evidence of the diversity, ubiquity, and significance of microbiomes has shifted attention from the study of bacteria in pure culture to the complex environments of multispecies communities (1). Beyond a fundamental understanding of communities lies the possibility of manipulating them to improve environmental, agricultural, and human health (2). However, the complexity of microbiomes has stymied attempts to alter them predictably and reliably. To fully leverage the practical benefits of microbial communities, we must uncover the mechanisms of interspecies interactions. Dissecting interactions among the dozens, hundreds, or thousands of species presents new challenges; the very complexity that makes microbiomes intriguing makes them difficult to study. Genetic and biochemical methods that have revealed the physiology and behavior of bacteria in pure culture are more difficult in complex communities. Thus, simple model microbiomes provide an appealing tool for establishing the principles that govern community behavior (3–6).

Simplified, “synthetic” communities can be constructed from microorganisms isolated from the same habitat but are not necessarily known to interact in their natural setting. Synthetic communities containing three or more species have been developed to study biofilm formation (7, 8), bacterial growth (9–11), and metabolism (12–14) from environments, including soil (9, 13), freshwater (12), the human oral microbiome (8), and the gut microbiome (10, 11, 14). Another type of simplified system is the “model community,” constructed from organisms known to interact in their original habitat. Examples of model communities with three or more species isolated from the rhizosphere (15), agriculture (15, 16), fermented foods (17–19), wound infections (20), and industrial microbiology (21) have been developed to dissect bacterial growth interactions (15, 17–20), metabolism (21), biofilm formation (15, 16), and antibiotic production (22).

Community-wide profiles provided by -omics tools are beginning to illuminate the signals mediating multilayered interspecies interactions (14, 23). Just as transcriptomic analysis has transformed our understanding of gene expression networks in bacteria living in pure culture, metatranscriptomics offers insight into functional properties of intact communities (24, 25). In a simple model microbiome, comprehensive metatranscriptomic changes can be quantified as each species encounters stimuli from others, providing the basis for mapping combinatorial interspecies interactions.

We previously described THOR (The Hitchhikers of the Rhizosphere), a three-species model microbiome constructed with members that appear to interact in their natural system (15). All members are genetically tractable so that mutant analyses can be applied to the community setting. The organisms in THOR are sourced from roots of field-grown plants. The cornerstone member is Bacillus cereus UW85, originally discovered on an alfalfa root (26). B. cereus UW85 colonizes and suppresses root diseases of both soybeans and alfalfa (26, 27). While isolating B. cereus strains from field-grown soybean roots, a curious phenotype appeared. Over several locations and years, 4 to 5% of colony-purified B. cereus coisolated with another bacterium that appeared after several weeks at 4°C. The coisolates, or “hitchhikers,” were strongly enriched (80%) for members of the *Bacteroidetes*, in particular, members of the *Cytophaga*-*Flavobacterium* group (28). The tight association of the hitchhikers with B. cereus and the apparent taxonomic enrichment hinted at physical and/or biological interactions in the field.

THOR’s three members display several striking interactions in pairs and as an entire community. B. cereus releases fragments of peptidoglycan that enable *F. johnsoniae* to grow in root exudate (28). The third member of THOR, Pseudomonas koreensis, produces the antibiotic koreenceine, which inhibits growth of *F. johnsoniae* (22). B. cereus protects *F. johnsoniae* from koreenceine, demonstrating an emergent community property—one that cannot be predicted from individuals or pairwise interactions (22). B. cereus and *F. johnsoniae*, individually and in tandem, enhance biofilm formation by *P. koreensis* (15). The other members induce dendritic, spreading colonies in B. cereus (15). Despite all these interactions, the mechanisms underpinning these phenotypes and the network of transcriptional response between THOR species remain unknown. To map the full scope of interspecies interactions, we generated transcriptomic profiles of individual species, pairs, and the entire community to identify genes important for interactions in community life. This analysis enabled us to determine whether the community profile is a sum of pairwise interactions or displays patterns of community-specific gene expression. We predicted that if there are community-specific genes, they would likely be unstudied because identification of gene function has been conducted largely in pure culture in which such genes may not display a function.

## RESULTS

**FIG 1:**
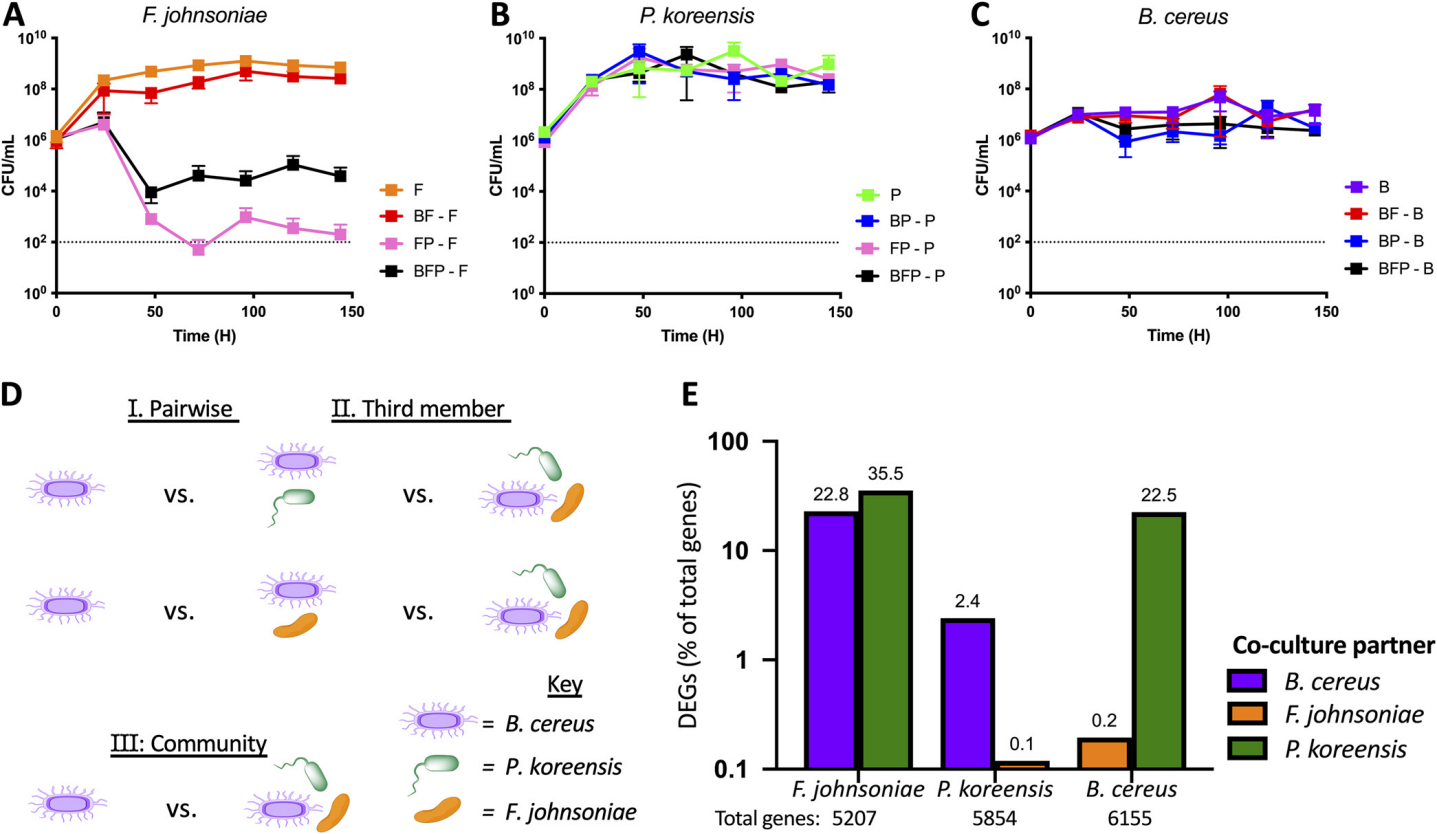
THOR member with coculture fitness defect exhibits greatest transcriptional response. Each THOR member was inoculated at 1 × 10^6^ CFU/mL alone, in pairwise coculture and full community. Every 24 h, cultures were quantified by dilution plating on species-specific antibiotics to determine CFU/mL levels of *F. johnsoniae* (A), *P. koreensis* (B), and B. cereus (C) under the different coculture conditions over 6 days. Data are shown as biological and technical duplicates. Dotted horizontal lines indicate the limit of detection. (D) Schematic of comparisons to uncover differential expression changes in response to pairwise coculture (category I), the addition of the third THOR member (category II), and the full community (category III). A comparison of the B. cereus conditions is shown as an example. (E) Global pairwise expression changes (>2-fold) are shown as a percentage of the total number of genes within each species.

The Hitchhikers of the Rhizosphere (THOR) is a model community composed of three microorganisms: Pseudomonas koreensis, Bacillus cereus, and Flavobacterium johnsoniae. In cultures initially containing equal abundance of each species in single, pairwise, or the three-species combinations (Fig. 1A to C), *P. koreensis* became the most abundant member. *P. koreensis* reduced growth of *F. johnsoniae* when they were grown as a pair, but *F. johnsoniae* abundance was higher when B. cereus was also present (Fig. 1A, black versus pink). This result is consistent with the ability of B. cereus to reduce production of the antibiotic koreenceine by *P. koreensis*, thereby protecting *F. johnsoniae* (15, 22). In contrast, the growth of *P. koreensis* and B. cereus was not affected by coculture (Fig. 1B and C, respectively).

To detect the transcriptional consequence of signaling that might govern the interactions within THOR and capture adequate sequence reads from the members of THOR whose abundance is low, we selected the early time point of 19.5 h to harvest RNA (see Fig. S1A) and sequenced transcripts of pure cultures and pairwise and triple cocultures (see Fig. S1B). For simplicity, the three-species condition will be referred to as the “community.” By comparing the level of each species’ transcripts between single, pairwise, and community conditions, we quantified gene expression changes in each species as it encountered increasing microbial diversity.

10.1128/mBio.02486-21.4 FIG S1 Cell density of THOR community combinations for RNA-Seq. (A) The THOR community was inoculated together in equal numbers (1 × 10^6^ CFU/mL) in biological duplicate. At the indicated time points, cultures were subjected to dilution plating on species-specific antibiotics to determine cell density of B. cereus (purple), *F. johnsoniae* (orange), and *P. koreensis* (green). The time point selected for RNA-Seq is shown by a dashed line (19.5 h). (B) Cell density (CFU/mL) of samples submitted for RNA-Seq for the wild-type conditions. (C) Cell density (CFU/mL) of samples submitted for RNA-Seq for the Δ*kec* conditions. Download FIG S1, TIF file, 61.2 MB. Copyright © 2022 Hurley et al. 2022 Hurley et al. https://creativecommons.org/licenses/by/4.0/ This content is distributed under the terms of the Creative Commons Attribution 4.0 International license.

### Unique pairwise transcriptional responses for each member of THOR.

The average number of reads was comparable for each THOR member alone: 2.3 million for *P. koreensis* (P), 4 million for B. cereus (B), and 1.7 million for *F. johnsoniae* (F). In the coculture samples, *P. koreensis* displayed transcript numerical dominance. For all coculture conditions, *P. koreensis* averaged 17.5 million gene-mapped reads, B. cereus averaged 2.9 million, and *F. johnsoniae* averaged 0.4 million. Mapped reads for genes above a low expression threshold of 10 reads across all four conditions (alone, two pairwise, and community) represent 66.8% of *P. koreensis* (3,908/5,854), 57.6% of B. cereus (3,548/6,155), and 77.9% of *F. johnsoniae* (4,054/5,207) genes. For each species, five comparisons were made between the four conditions. An example for B. cereus is shown in Fig. 1D. Transcript levels of B. cereus alone compared to B. cereus in either two-species condition revealed genes important for pairwise coculture, designated category I or “pairwise” genes. Comparison of transcript levels of B. cereus in a pairwise condition with the levels in the community assessed the influence of either *P. koreensis* or *F. johnsoniae* as the third member, designated category II or “third member” genes. Comparison of transcript levels of B. cereus alone and in the community, designated category III, or community genes, revealed the influence of both *F. johnsoniae* and *P. koreensis* together on B. cereus. All differentially expressed genes (DEGs) shown in the following analysis have a false discovery rate (FDR) adjusted *P* value of <0.05. Regulation is generally defined as a gene expression change of >2-fold (1 in log_2_). Volcano plots of category I to III interactions for each species are displayed in Fig. S2.

10.1128/mBio.02486-21.5 FIG S2 Volcano plots of THOR RNA-Seq comparisons. The log_2_-fold change (FC) of DEGs on the *x* axis compared to their log_10_ *P* value on the *y* axis for pairwise or category I (A to F), third member or category II (G to K), and community or category III (L to N) comparisons. Upregulated DEGs greater than the vertical line at log_2_ FC = 1 shown in green and downregulated DEGs less than the vertical line at log_2_ FC = −1 shown in blue. Panels are organized in columns by species and the range of axis change based on the data. Condition comparisons are presented as follows. (A) F versus BF, (B) B versus BF, (C) P versus BP, (D) F versus FP, (E) B versus BP, (F) P versus FP, (G) FP versus BFP for F DEGs, (H) BP versus BFP for B DEGs, (I) FP versus BFP for P DEGs, (J) BF versus BFP for F DEGs, (K) BF versus BFP for B DEGs, (I) F versus BFP, (M) B versus BFP, (N) P versus BFP. There were no significant category II DEGs identified in the BP versus BFP comparison. Download FIG S2, TIF file, 95.4 MB. Copyright © 2022 Hurley et al. 2022 Hurley et al. https://creativecommons.org/licenses/by/4.0/ This content is distributed under the terms of the Creative Commons Attribution 4.0 International license.

The starkly different communication strategies of the THOR members became apparent by comparing pairwise interactions. The number of DEGs regulated >2-fold for each species in pairwise culture with the other two THOR members is shown in Fig. 1E as a percentage of the total genes in each species. *F. johnsoniae* was responsive to the THOR community, dramatically modulating gene expression upon addition of either member. *F. johnsoniae* changed expression of 22.8 and 35.5% of its genes in response to either B. cereus or *P. koreensis*, respectively. *P. koreensis*, in contrast, responded minimally, regulating 2.4% of its genes in response to B. cereus, and only 0.1% (seven genes) were differentially regulated in response to coculture with *F. johnsoniae*. *P. koreensis* was the least responsive member of THOR and the most influential, altering expression of about one-quarter and one-third of B. cereus and *F. johnsoniae* genes, respectively. B. cereus responded strongly to *P. koreensis* and weakly to *F. johnsoniae*, indicating that the strength of its transcriptional response was intermediate between those of the other two community members (Fig. 1E).

### Single-species DEGs are more abundant than those regulated by both partners.

**FIG 2:**
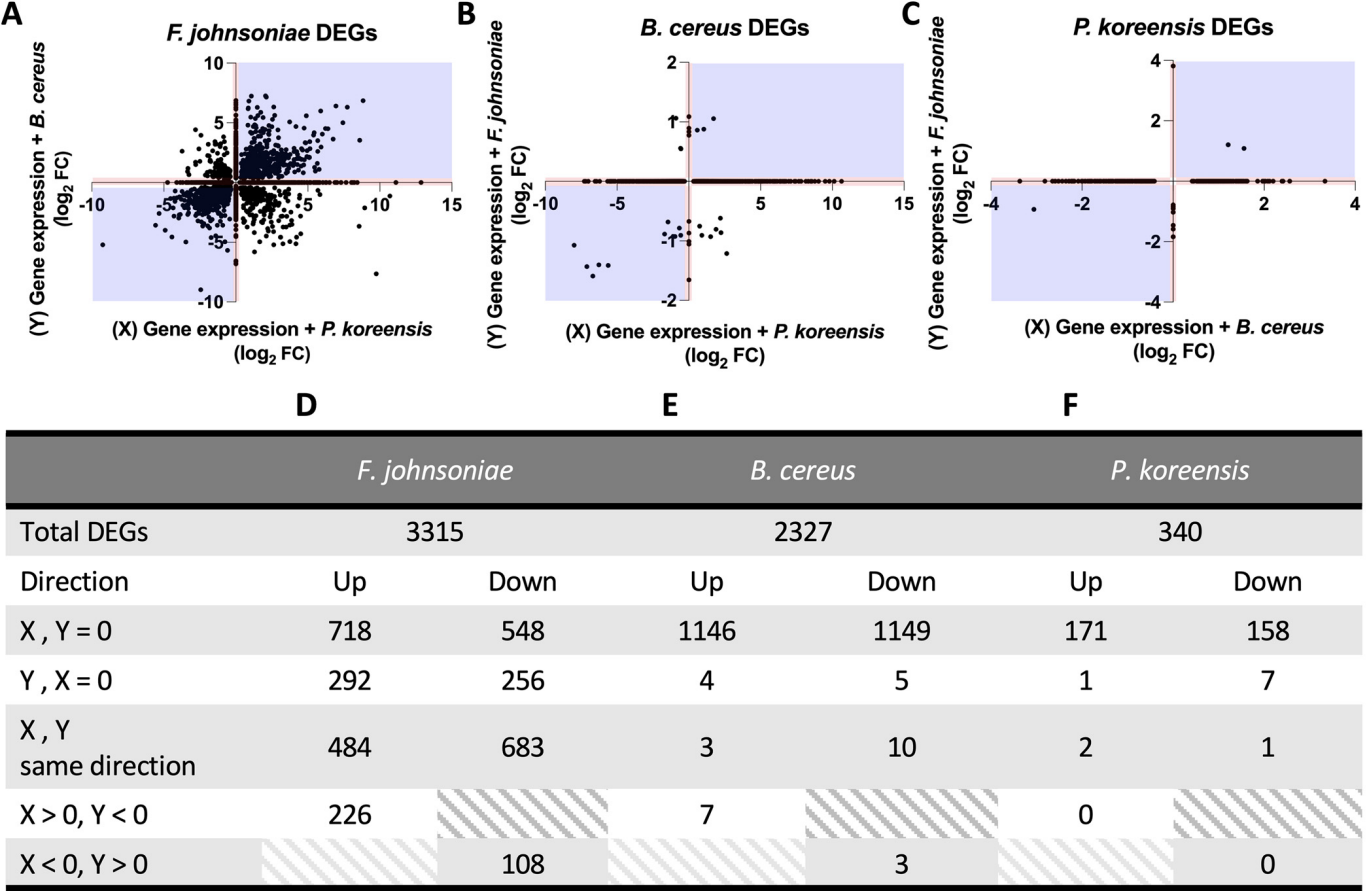
Single-species responses dominate THOR transcriptional regulation. (A) For each gene in *F. johnsoniae* with statistically significant category I regulation, the log_2_-fold change in coculture with *P. koreensis* was plotted on the *x* axis, with the log_2_-fold change in coculture with B. cereus plotted on the *y* axis. There was no fold change cutoff implemented; all statistically significant differentially expressed genes were included in the analysis. Points along the axes indicate genes that were only regulated in the presence of a single partner. *F. johnsoniae* differentially expressed many genes in response to either THOR member in both the same (blue quadrants) and opposite (white quadrants) regulation patterns. Pairwise coculture data were similarly displayed for B. cereus (B) and *P. koreensis* (C). The total number of genes regulated and their regulatory pattern breakdowns are shown in the table columns for *F. johnsoniae* (D), B. cereus (E), and *P. koreensis* (F).

*P. koreensis* and B. cereus largely responded to each other’s presence but not to *F. johnsoniae*, whereas *F. johnsoniae* modulated a large proportion of its genome in response to either partner (Fig. 1E). We next sought to determine, especially in the case of *F. johnsoniae*, the extent to which genes were regulated by a single species or by both species. By comparing the expression level, in log_2_-fold change, of a DEG in response to one pairwise coculture partner versus the other pairwise coculture partner, the direction and extent of regulation can be visualized to determine whether the DEG is regulated by one or both coculture partners (Fig. 2). There was no fold change cutoff implemented; all statistically significant differentially expressed genes were included for a global analysis.

In all three members, a single-species response to either *P. koreensis* or B. cereus dominated the pairwise transcriptional regulation, as illustrated by the majority of points along the *x* axis in the scatterplots in Fig. 2A to C and in the number of DEGs in the tables shown in Fig. 2D to F. *F. johnsoniae* showed the most heterogenous gene expression patterns among the different conditions. Hundreds of genes responded exclusively to the presence of *P. koreensis* (Fig. 2A and x-axis) or B. cereus (Fig. 2A and y-axis). In addition, one third of *F. johnsoniae* DEGs were regulated in the same direction (both up or both down) by *P. koreensis* and B. cereus (Fig. 2A, blue quadrants). The largest subset of downregulated genes in *F. johnsoniae* were downregulated in the presence of either partner (Fig. 2D, 683 genes) and the largest subset of upregulated genes in *F. johnsoniae* were upregulated in the presence of *P. koreensis* only (Fig. 2D, 718 genes). Of all three THOR members, *F. johnsoniae* revealed the largest proportion of opposing responses (upregulated by one and downregulated by the other) to the other two members (Fig. 2A, white quadrants).

### Dual-species response is nonadditive in the community.

**FIG 3:**
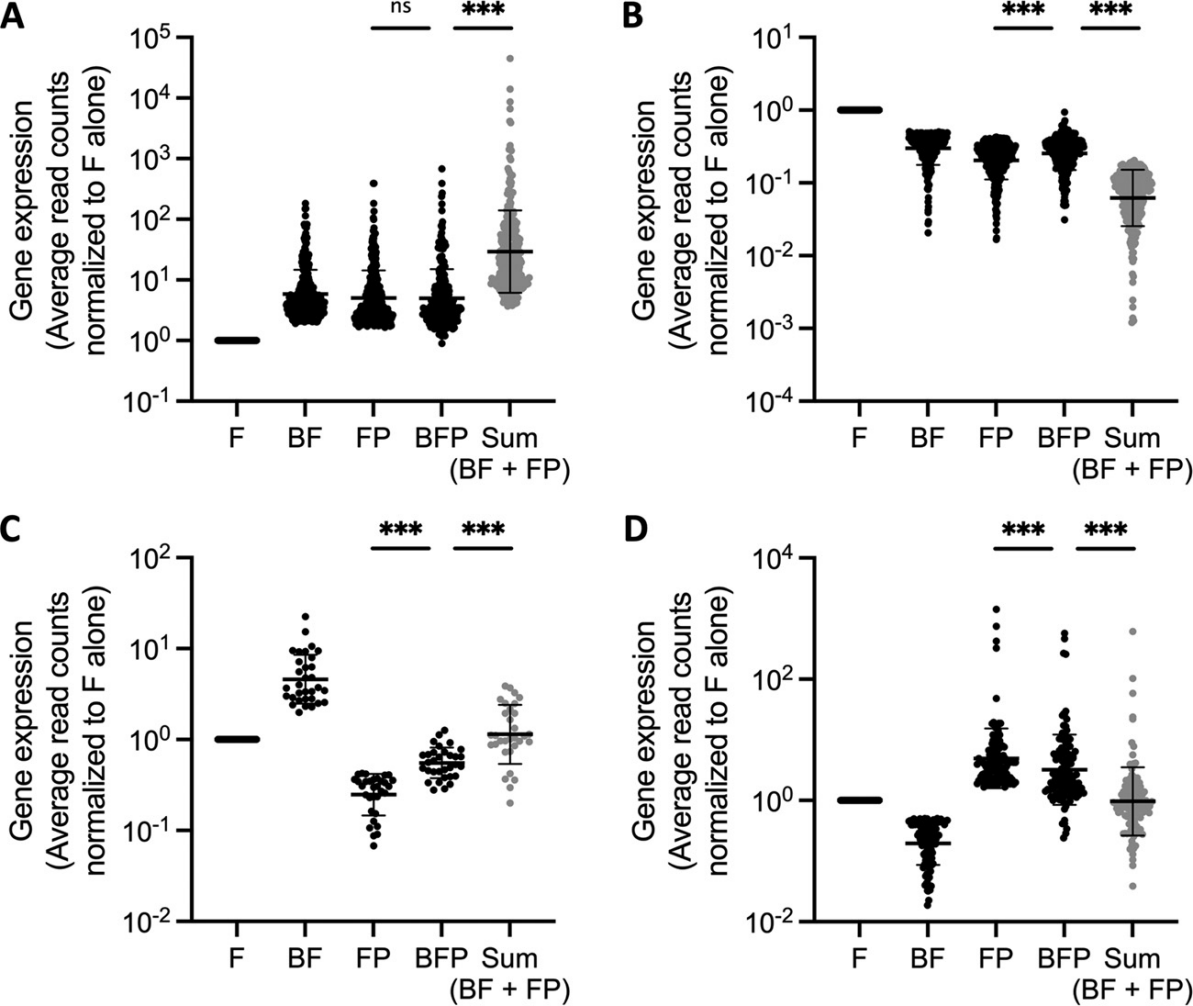
Dual-species responses in *F. johnsoniae* are nonadditive with *P. koreensis* dominating opposite responses in the community setting. F = *F. johnsoniae*, B = *B. cereus*, and P = *P. koreensis*. DEG normalized transcript levels for the replicates from edgeR were averaged in each condition. Then, pairwise and community conditions were each divided by expression levels of the species alone. The data are displayed as populations on a log_10_ scale. (A and B) Positively (A) and negatively (B) regulated DEGs in pairwise coculture >2-fold with B. cereus and *P. koreensis* (Fig. 2A, blue quadrants) were also regulated in the community. (C and D) *F. johnsoniae* expression of genes regulated in opposite directions by B. cereus and *P. koreensis* (Fig. 2A, white quadrants) in the community exhibited levels similar to coculture with *P. koreensis*. The measured BFP values differ from the predicted “Sum” (gray), which was calculated for each DEG to determine whether community expression level was a result of the additive effect of both pairwise interactions. All significance was determined by a paired, nonparametric Wilcoxon test with a Bonferroni correction. ***, *P* < 3.03 × 10^−5^.

For genes in *F. johnsoniae* regulated by both species in pairwise coculture, we next quantified the gene expression changes exhibited in the community condition, written as BFP to indicate the presence of all three species (B, F, and P). If there was an additive or synergistic response, the DEGs should display greater expression levels in the community compared to the two pairwise conditions. The DEGs that responded to B. cereus and *P. koreensis* in the same direction (blue quadrants in Fig. 2A, >1-log_2_ fold change [FC] with each partner) displayed expression levels in the community no greater than the pairwise conditions (Fig. 3A and B). To highlight the nonadditive property of dual-species transcriptional response in *F. johnsoniae*, the sum of both pairwise interactions was calculated and compared to the measured community condition (Fig. 3, complete community versus Sum). For *F. johnsoniae* genes with opposite directions of regulation between THOR coculture partners (white quadrants in Fig. 2A, >1 and <–1-log_2_ FC with either partner), the community level should have centered around 0 on the *y* axis based on the additive calculations of the pairwise levels (Fig. 3C and D, Sum), but we found the overall community condition was closer to the degree of change seen in the coculture with *P. koreensis*, hinting at the dominant role of *P. koreensis* in the transcriptional response of the community.

### Pairwise DEGs in response to *P. koreensis* are also regulated in the community.

**FIG 4:**
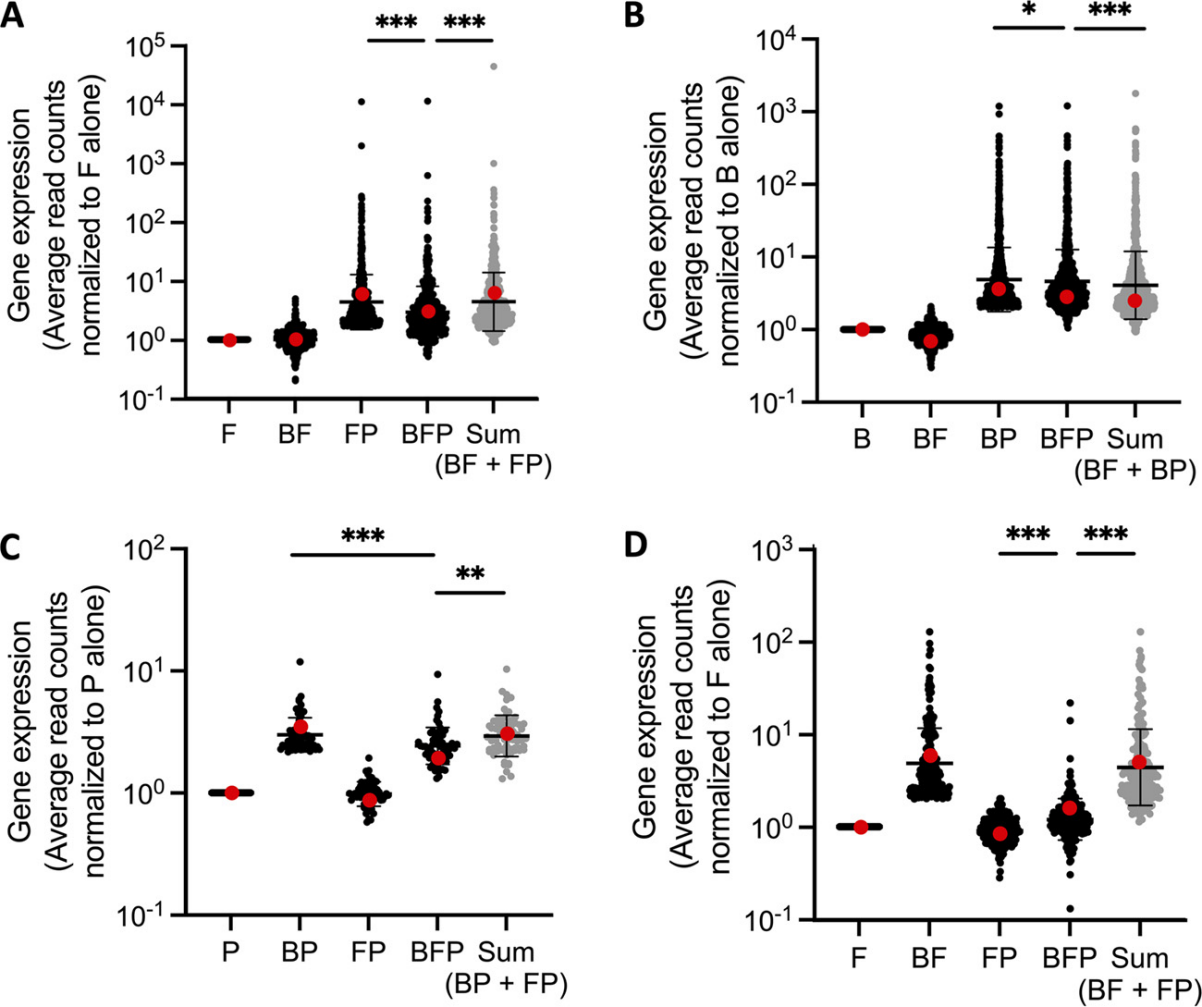
Single-species responses to *P. koreensis* and B. cereus differ in the community setting. See the Fig. 3 legend for a DEG normalization description and Sum calculation. (A) *F. johnsoniae* upregulated genes >2-fold in response to *P. koreensis* (Fig. 2A and >1-log2 *x* axis) were also upregulated in the community. (B) B. cereus upregulated genes >2-fold in response to *P. koreensis* (Fig. 2B and >1-log2 *x* axis) were also upregulated in the community. (C) *P. koreensis* upregulated genes >2-fold in response to B. cereus (Fig. 2C and >1-log2 *x* axis) were also upregulated in the community. (D) *F. johnsoniae* upregulated genes >2-fold in response to B. cereus (Fig. 2A and >1-log2 *y* axis) demonstrated reduced regulation in the community. Representative genes (red) in each panel highlight overall trends, specifically, the expression levels in BFP did not match the dominant pairwise or the calculated pairwise sum. (A) FJOH_RS08820 *clpP*, (B) A9L49_RS16845 *kinB*, (C) BOW65_RS13585 putative oxidoreductase, (D) FJOH_RS04840 hypothetical protein. All significance was determined by a paired, nonparametric Wilcoxon test with a Bonferroni correction. *, *P* = 9.69 × 10^−4^; **, *P* = 1.54 × 10^−4^; ***, *P* < 2.26 × 10^−10^.

For single-species regulation (DEGs on the axes of Fig. 2), we sought to determine whether they were similarly regulated in the community. For simplicity, the focus will be on upregulated genes. We found that all *F. johnsoniae* DEGs only upregulated by *P. koreensis* in pairwise coculture (Fig. 2A, >1-log_2_ *x* axis) were also upregulated in the community (Fig. 4A, BFP). Similarly, all B. cereus DEGs upregulated only by *P. koreensis* in pairwise coculture (Fig. 2B, >1-log_2_ *x* axis) were also upregulated in the community (Fig. 4B, BFP). However, we found the community mean expression level of the *P. koreensis*-specific DEGs to be less than the mean expression level observed in the pairwise coculture for both *F. johnsoniae* (Fig. 4A, FP versus BFP) and B. cereus (Fig. 4B, BP versus BFP). We first hypothesized the difference between the overall pairwise and community expression levels could be due to subtle pairwise interactions with the third THOR member. Thus, we calculated the sum of both pairwise interactions as we had in the dual-species response analysis. The pairwise sum of *P. koreensis*-specific upregulated DEGs in both *F. johnsoniae* (Fig. 4A, BFP versus Sum) and B. cereus (Fig. 4B, BFP versus Sum) were statistically different from the levels measured in the community, as measured by the Wilcoxon test with a Bonferroni correction. Representative genes (FJOH_RS08820 *clpP* and A9L49_RS16845 *kinB*) demonstrated the general trend of non-additive, reduced community expression (Fig. 4A and B, red values). Such trends were also found in pairwise *P. koreensis*-specific downregulated DEGs (see Fig. S3A and B).

10.1128/mBio.02486-21.6 FIG S3 Downregulated single-species DEGs show similar trends to upregulated DEGs. DEG normalized transcript levels for the replicates from edgeR were averaged in each condition. Then, pairwise and community conditions were each divided by expression levels of the species alone. The data are displayed as populations on a log_10_ scale. The measured BFP values differ from the predicted “Sum” (gray), which was calculated for each DEG to determine whether community expression level was a result of the additive effect of both pairwise interactions. (A) *F. johnsoniae* DEGs downregulated >2-fold in response to *P. koreensis* (Fig. 2A, <0 *x* axis) were also downregulated in the community. (B) B. cereus DEGs downregulated >2-fold in response to *P. koreensis* (Fig. 2B, <0 *x* axis) were also downregulated in the community. (C) The only setting in which expression levels in the community were indistinguishable from the pairwise coculture was *P. koreensis* DEGs downregulated >2-fold in response only to B. cereus in pairwise coculture (Fig. 2C, <0 *x* axis). (D) *F. johnsoniae* DEGs downregulated >2-fold in response to B. cereus (Fig. 2A, <0 *y* axis) demonstrated reduced regulation in the community. All significance was determined by a paired, nonparametric Wilcoxon test with a Bonferroni correction. ***, *P* < 1.42 × 10^5^. Download FIG S3, TIFF file, 26.3 MB. Copyright © 2022 Hurley et al. 2022 Hurley et al. https://creativecommons.org/licenses/by/4.0/ This content is distributed under the terms of the Creative Commons Attribution 4.0 International license.

After ruling out additive pairwise interactions with the neutral partner, we next considered that there may be higher-order influence of the pairwise-neutral partner, either directly or indirectly, on the *P. koreensis*-specific DEGs when in the community. If so, we should find, for example, category II regulation by B. cereus within the *P. koreensis-*specific DEGs in *F. johnsoniae*. While the species-specific DEGs had a >2-fold change cutoff, the higher-order interactions were not filtered by expression cutoffs to capture subtle effects. Indeed, we found higher-order interactions by B. cereus on 242 of 478 *P. koreensis*-specific upregulated DEGs in *F. johnsoniae* (see Data Set S3, tab 1). Similarly, *F. johnsoniae* also exhibited higher-order interactions on 56 of 748 *P. koreensis*-specific upregulated DEGs in B. cereus (see Data Set S3, tab 2). Although the category II *F. johnsoniae* effect on B. cereus genes regulated by *P. koreensis* was smaller, the overall effect of *F. johnsoniae* on B. cereus was greater as a community member rather than as a pairwise coculture partner. As seen in Fig. 2E, only 4 B. cereus genes total are exclusively upregulated by *F. johnsoniae*.

10.1128/mBio.02486-21.3 DATA SET S3 Category II. The gene IDs, functions, and log_2_-fold changes for DEGs with an FDR *P* value of <0.05 are shown for *F. johnsoniae* DEGs regulated >2-fold in response to only *P. koreensis* in pairwise (tab 1), B. cereus DEGs regulated >2-fold in response to only *P. koreensis* in pairwise (tab 2), and *F. johnsoniae* DEGs regulated >2-fold in response to only B. cereus in pairwise (tab 3). The presence of regulation by the third member was noted in column H. Download Data Set S3, XLSX file, 0.2 MB. Copyright © 2022 Hurley et al. 2022 Hurley et al. https://creativecommons.org/licenses/by/4.0/ This content is distributed under the terms of the Creative Commons Attribution 4.0 International license.

### Regulation of *B. cereus*-specific DEGs in the community depends on *P. koreensis*.

*P. koreensis* DEGs upregulated by only B. cereus in pairwise coculture (Fig. 2C, >1-log_2_ *x* axis) were also upregulated in the community (Fig. 4C, BP versus BFP). The representative gene expression of the putative oxidoreductase BOW65_RS13585 displays the general trend of nonadditive, reduced community expression (Fig. 4C, red values). For *P. koreensis* DEGs downregulated by B. cereus, the downregulation observed in the community was indistinguishable from the expression level in the pairwise condition (see Fig. S3C, BP versus BFP), suggesting *F. johnsoniae* had no higher-order influence on *P. koreensis* DEGs downregulated by B. cereus.

In contrast, *F. johnsoniae* DEGs upregulated by only B. cereus in pairwise (Fig. 2A, >1-log_2_ *y* axis), were barely upregulated, if at all, in the community (Fig. 4D, BF versus BFP). In fact, the mean community expression level of B. cereus-specific DEGs resembled the level seen in coculture with *P. koreensis* (Fig. 4D FP versus BFP), as if the presence of *P. koreensis* prevented regulation of the B. cereus*-*specific DEGs in the community. Category II regulation by *P. koreensis* occurred in the majority (174 of the 206) of *F. johnsoniae* DEGs upregulated by B. cereus in pairwise culture, showing that higher-order regulation by P. koreensis is the norm (see Data Set S3, tab 3). *F. johnsoniae* DEGs downregulated by only B. cereus in the pairwise condition showed a similar pattern: strongly reduced regulation in the community condition (see Fig. S3D). In the community, B. cereus-specific pairwise gene regulation in *F. johnsoniae* is largely muted by the presence of *P. koreensis*, again suggesting *P. koreensis* plays a dominant role in the transcriptional response of the community.

### *P. koreensis* pairwise interactions drive community response.

Given the dominance of *P. koreensis* in both single-species and dual-species responses in the other two THOR members, we quantified how well the *P. koreensis* pairwise (Fig. 1D, category I) and the third member (Fig. 1D, category II) comparisons explain the gene expression changes in the community (Fig. 1D, category III). We found the strongest log_2_-linear relationship between the fold change of category III DEGs and the corresponding fold change in category I *P. koreensis* pairwise coculture for both B. cereus (see Fig. S4A, *R*^2^ = 0.9736) and *F. johnsoniae* (see Fig. S4B, *R*^2^ = 0.912).

10.1128/mBio.02486-21.7 FIG S4 Pairwise interactions with *P. koreensis* drive community response in B. cereus and *F. johnsoniae*. (A) Category III DEG log_2_-fold change (FC) for B. cereus shown on the *x* axis with DEG log_2_ FC for that gene in pairwise coculture with *P. koreensis* (dark green), in pairwise coculture with *F. johnsoniae* (orange), with *P. koreensis* as the third member (light green), and with *F. johnsoniae* as the third member (blue) on the *y* axis. (B) Category III DEG log_2_ FC for *F. johnsoniae* shown on the *x* axis with DEG FC for that gene in pairwise coculture with *P. koreensis* (dark green), in pairwise coculture with B. cereus (purple), with *P. koreensis* as the third member (light green), and with B. cereus as the third member (pink) on the *y* axis. Best-fit line, slope, and *R*^2^ values are shown for each linear relationship between the FC in category III with the respective FC seen in category I or II. Download FIG S4, TIF file, 20.5 MB. Copyright © 2022 Hurley et al. 2022 Hurley et al. https://creativecommons.org/licenses/by/4.0/ This content is distributed under the terms of the Creative Commons Attribution 4.0 International license.

### Koreenceine coordinates *P. koreensis* pairwise interactions.

**FIG 5:**
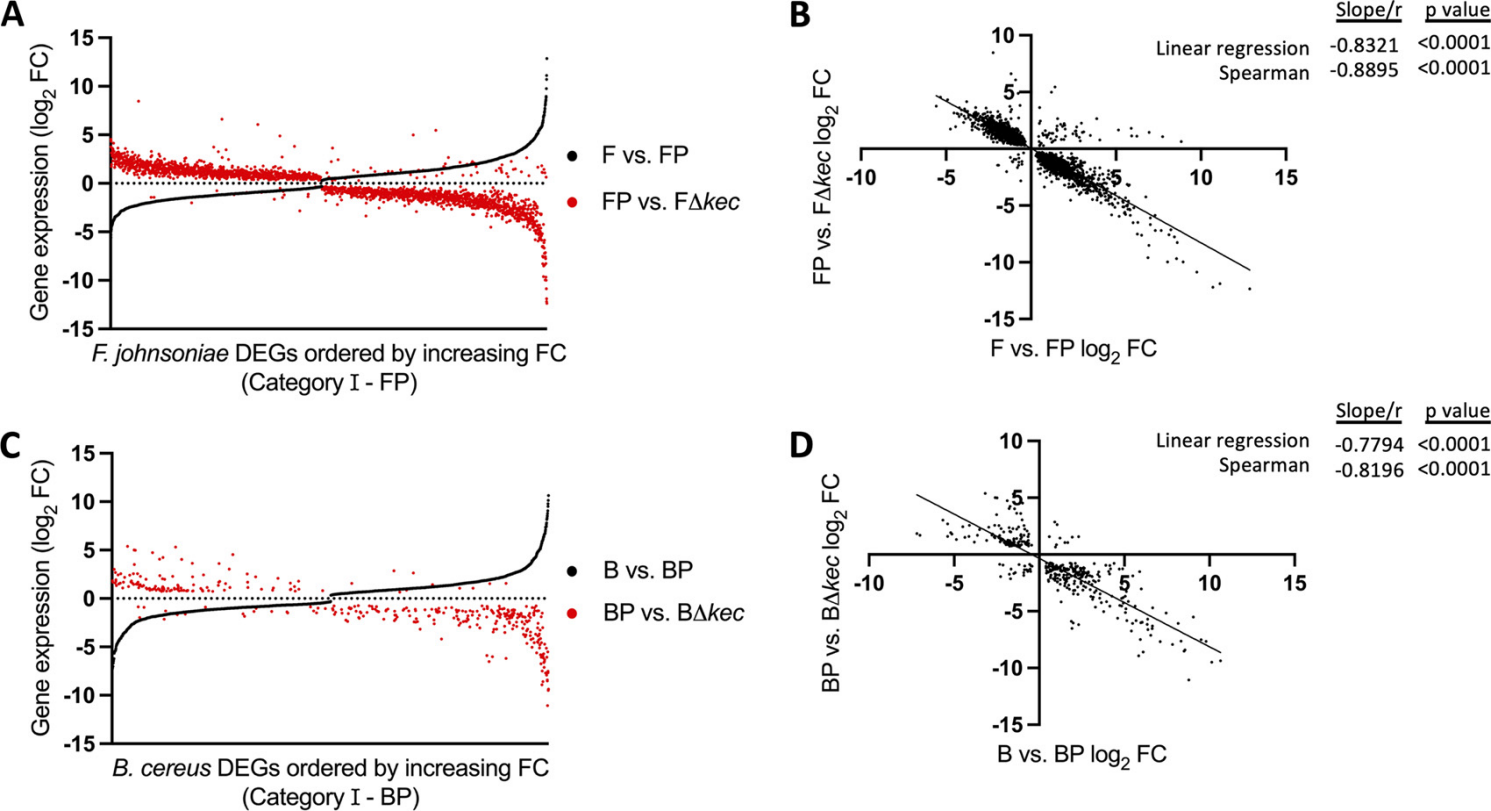
Loss of koreenceine reverses direction of gene regulation in pairwise coculture with *P. koreensis*. (A) *F. johnsoniae* DEGs in pairwise with *P. koreensis*, both single- and dual-species categories, aligned with increasing fold change (black) on the *x* axis. The comparable fold change (FC) for that gene in the wild-type FP pairwise compared to the FΔ*kec* pairwise shown in red. (B) Linear regression and Spearman correlation between log_2_ FC of F versus FP pairwise DEGs against FP versus FΔ*kec.* (C) B. cereus genes differentially expressed in pairwise with *P. koreensis* aligned with increasing FC (black) on the *x* axis. The FC for that gene in the wild-type BP pairwise compared to the BΔ*kec* pairwise shown in red. (D) Linear regression and Spearman correlation between log_2_ FC of B versus BP pairwise DEGs against BP versus BΔ*kec*.

*P. koreensis* was the most abundant member in the THOR community and its secondary metabolite, koreenceine, inhibits *F. johnsoniae* growth (22). We hypothesized koreenceine mediates *P. koreensis* relative abundance and and thus its signaling dominance. To quantify the impact of koreenceine on THOR signaling, we conducted RNA-Seq on *P. koreensis* alone, in pairwise coculture with B. cereus or *F. johnsoniae* and in the community using either the wild-type strain or a *P. koreensis* mutant (22) that does not produce koreenceine, a deletion mutant of the koreenceine biosynthetic gene cluster, *kecABCDEFGHIJK*; referred to henceforth as Δ*kec* (see Fig. S1C). *F. johnsoniae* DEGs whose expression changed >2-fold in response to wild-type *P. koreensis* were ordered by increasing fold change in Fig. 5A (black). We found that most genes were also regulated in response to coculture with the Δ*kec* mutant (Fig. 5A, red), but the direction of observed changes was largely opposite. Genes that were downregulated by the wild type were upregulated compared to the mutant and vice versa. 85% of *F. johnsoniae* genes with >2-fold regulation in the wild-type pairwise coculture (1,579/1,850 genes) can be explained by presence of the koreenceine biosynthetic gene cluster (Fig. 5B, Spearman correlation −0.8895), likely a combination of direct effects from koreenceine and indirect effects due to slower growth rate of *F. johnsoniae* in the presence of an antibiotic. An alternative hypothesis is that these genes were cell density regulated since *F. johnsoniae* was 100-fold more abundant in coculture with the Δ*kec* mutant than with the wild type. However, the *F. johnsoniae* genome does not contain common quorum-sensing autoinducer genes—neither acyl-homoserine lactone synthase (EC 2.3.1.184) nor *luxS* (EC 4.4.1.21) homologues. A novel quorum-sensing system in the *Bacteroidetes* phylum might await discovery, but our current hypothesis is that these genes (Fig. 5A and B) were likely regulated in response to *P. koreensis* coculture and not *F. johnsoniae* cell density.

B. cereus displayed a similar response to the Δ*kec* mutant (Fig. 5C). The direction of expression of B. cereus DEGs was opposite in the wild type and Δ*kec* mutant. However, the proportion of genes regulated by koreenceine in B. cereus was less than in *F. johnsoniae*, as shown by the comparatively sparse red symbols in Fig. 5C. Only 22% of genes in B. cereus (303/1383) with >2-fold regulation in the pairwise coculture can be explained by the presence of koreenceine (Fig. 5D, Spearman correlation −0.8196). Overall, in pairwise coculture with *P. koreensis*, koreenceine mediated the majority of gene expression changes in *F. johnsoniae* and also induced significant gene expression changes in B. cereus, even though B. cereus growth was unaffected.

### Functional response to koreenceine is largely different between THOR members.

**FIG 6:**
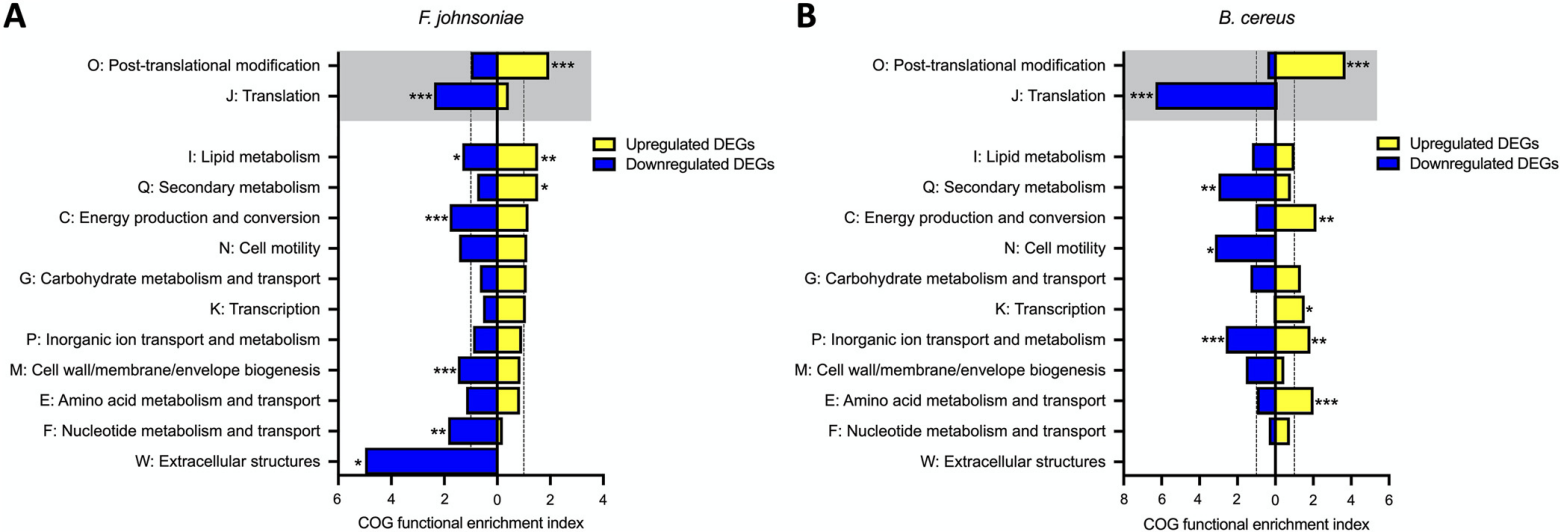
Koreenceine elicits both conserved and species-specific responses. *F. johnsoniae* and B. cereus genomes were functionally annotated using eggNOG-mapper and the baseline prevalence of each COG category was quantified. Koreenceine-dependent genes sets were identified as >2-fold *P. koreensis*-regulated genes with opposing direction of regulation in the Δ*kec* coculture (see Fig. 5). COG functional enrichment index was quantified as the percentage of COG category present in koreenceine-dependent DEGs divided by the percentage of that particular COG category in the whole genome. To be enriched, the functional annotation must have an index of >1 (dotted lines), meaning the frequency of that function in the data set is greater than found in the genome. Functional enrichment of both upregulated (yellow) and downregulated (blue) DEGs were analyzed for *F. johnsoniae* (A) and B. cereus (B). Only COG categories with at least one significant enrichment between the two species are shown. COG categories shaded gray are enriched in the same direction in both species. Significance was determined in Rstudio by Fisher exact test (*, *P < *0.05; **, *P < *0.01; ***, *P < *0.001).

The strong responses to koreenceine in both *F. johnsoniae* and B. cereus led us to ask whether the responses involved similar genes in the two species. We quantified the enrichment of gene functional categories in the DEGs mediated by koreenceine compared to the prevalence of that gene type in the genome for both *F. johnsoniae* and B. cereus. The koreenceine-dependent COG (clusters of orthologous groups) functional categories (29) enriched in both *F. johnsoniae* and B. cereus included upregulation in genes pertaining to posttranslational modification, protein turnover, and chaperones and downregulation for those involved in translation (Fig. 6, gray boxes).

To further characterize the shared koreenceine response, specific gene annotations for the up- and downregulated genes for both *F. johnsoniae* and B. cereus were compared. Of 831 *F. johnsoniae* and 196 B. cereus upregulated genes, there were 25 annotations shared between the two species’ koreenceine-induced gene expression (0.025 Jaccard similarity; genes shown in Table 1). The functions of the shared genes suggest that koreenceine induced stress in both species, although only *F. johnsoniae* populations decreased. Activation of *clpBCP*, *grpE*, *groL*, and *dnaJ* suggests protein damage occurred in both organisms (30, 31) and activation of superoxide dismutase (*sodA*) (32), *ohrA* (33), *ygiD* (34), *resA* (35), *ywnA* (36), and the putative *ytcD* (37) suggests the stress may be redox related. In addition, upregulation of heavy metal resistance genes (*arsBCR*, *arsR3*, *cadA*, Cu^+^ exporting ATPase) and general stress protein 18 further indicate a shared stress response (38, 39). Of 748 and 107 genes downregulated in *F. johnsoniae* and B. cereus, respectively, 20 shared annotations, of which 12 were related to translation (0.024 Jaccard similarity; genes shown in Table 2). Ribosomal proteins (*fusA*, *rpsL*, *rpsG*, *rpsU*, *rplM*, *rpsJ*, and *rplK*), tRNA biosynthesis genes (*valS*, *aspS*, *hisS*, and *proS*), and even methionine biosynthesis (*metE*) were all downregulated in both *F. johnsoniae* and B. cereus.

**TABLE 1:** Conserved koreenceine-dependent gene upregulation by *F. johnsoniae* and B. cereus indicates redox stress^*a*^

Gene ID	COG category	FC (log_2_)		Function
		B	F	
*arsB*	P	7.5	5.6; 5.1; 2.3	Arsenic resistance protein
*arsC*	T	6.4	6.3; 5.8; 3.2	Reduces arsenate As(V) to arsenite As(III)
*arsR*	K	6.7	4.3	Arsenial resistance operon repressor
*arsR3*	K	2.1	7.3	Arsenial resistance operon repressor
*asnB*	E	1.1	1.5	Asparagine synthase
*azoR*	I	5.6; 3.2	12.9	Reductive cleavage of azo bond in aromatic azo compounds
*cadA*	P	2.7	1.2	Zinc-exporting P-type ATPase
*clpB*	O	3.0	1.5	ATPase that targets misfolded proteins to ClpP proteinase
*clpC*	O	2.7	3.7	ATPase that targets misfolded proteins to ClpP proteinase
*clpP*	OU	3.1	2.9	Serine dehydrogenase proteinase
*dnaJ*	O	1.3	1.1	Chaperone
*grpE*	O	1.2	1.3	Chaperone
*groL*	O	1.3	1.2	Chaperone that prevents misfolding and promotes the refolding
*hisG*	E	2.8	1.8	First step of histidine biosynthesis
*namA*	C	2.6	1.1	Reduction of the double bond unsaturated aldehydes ketones and the nitro group of nitroester and nitroaromatic compounds; may have a role in detoxification processes
*sodA*	P	2.1	2.0	Superoxide dismutase
*tal*	G	2.0	1.2	Transaldolase important for the balance of metabolites in the pentose-phosphate pathway
*ygiD*	S	3.9	3.9	4,5-DOPA-extradiol-dioxygenase that produces antiradical betalains
*ywnA*	K	3.3	2.5	Redox-sensitive Rrf2-family regulator
General stress protein 18 (*ykfM*)	S	3.5	1.1	Stress response
Copper-exporting P-type ATPase	P	5.5	4.0; 4.0	Copper-exporting P-type ATPase
Organic hydroperoxide resistance protein OhrA	O	4.7	5.5	Reactive oxygen stress
Thiol-disulfide oxidoreductase ResA	CO	1.1	3.9; 2.9; 2.9; 1.7; 1.7; 1.7	Redox reactions via the reversible oxidation of an active center disulfide bond
Putative protein YhaZ	L	1.3	1.7	Similar to DNA alkylation repair COG4335 superfamily of proteins
Putative HTH-type transcriptional regulator YtcD	K	1.5	3.1	Reactive electrophile species MarR/DUF24-family regulator

a Gene annotations of koreenceine-dependent upregulated genes in *F. johnsoniae* (F) and B. cereus (B) were compared to identify genes present in both. The gene IDs, COG functional categories, log_2_-fold changes (FC) in pairwise coculture with *P. koreensis*, and functions for each shared gene are shown. Semicolons separate the FC of multiple genes with the same annotation.

**TABLE 2:** Conserved koreenceine-dependent gene downregulation by *F. johnsoniae* and B. cereus targets translation^*a*^

Gene ID	COG category	FC (log_2_)		Function
		B	F	
*aspS*	J	−1.4	−1.5	Aspartate-tRNA ligase
*atpF*	C	−1.2	−2.0	ATP synthase subunit b
*fusA*	J	−1.6	−2.0	Elongation factor G
*map*	E	−2.3	−1.4	Methionine aminopeptidase
*hisS*	J	−1.8	−1.4	Histidine-tRNA ligase
*metE*	E	−1.4	−2.3	Final step in methionine biosynthesis
*proS*	J	−2.3	−1.9	Proline-tRNA ligase
*rplK*	J	−1.7	−1.8	50S ribosomal protein L11
*rplM*	J	−1.7	−1.5	50S ribosomal protein L13
*rpsG*	J	−1.8	−1.8	30S ribosomal protein S7
*rpsJ*	J	−2.0	−1.4	30S ribosomal protein S10, involved in the binding of tRNA to the ribosomes
*rpsL*	J	−1.9	−1.8	30S ribosomal protein S12
*rpsU*	J	−1.7	−2.1	30S ribosomal protein S21
*secY*	U	−2.0	−1.9	Membrane protein translocation
*valS*	J	−2.0	−1.2	Valine-tRNA ligase
Peptidoglycan *O*-acetyltransferase	M	−1.1	−2.5; −1.6	Acetylates peptidoglycan to confer resistance to lysozyme and penicillin
Hemin transport system permease protein HmuU	P	−1.5	−1.4	Iron ABC transporter
Bacillolysin	E	−1.9	−1.8	Extracellular thermolysin metallopeptidase
tRNA threonylcarbamoyladenosine dehydratase	H	−1.4	−1.6	Molybdopterin and thiamine biosynthesis
Ferri-bacillibactin esterase BesA	S	−1.7	−1.3	Releases intracellular iron from siderophores

a Gene annotations of koreenceine-dependent downregulated genes in *F. johnsoniae* (F) and B. cereus (B) were compared to identify genes present in both. The gene IDs, COG functional categories, log_2_-fold changes (FC) in pairwise coculture with *P. koreensis*, and functions for each shared gene are shown. Semicolons separate the FC of multiple genes with the same annotation.

The gene with the strongest upregulation in all THOR species comparisons (Fig. 1D) was a NADH-dependent azoreductase (*azoR*, log_2_-fold change category I = 12.87 and III = 12.89) in *F. johnsoniae* in the presence of *P. koreensis.* Koreenceine-dependent *azoR* upregulation was also identified in B. cereus (Table 1). Furthermore, in *P. koreensis*, the Δ*kec* mutant showed a >270-fold downregulation in *azoR* compared to wild-type *P. koreensis*, suggesting that koreenceine activates *azoR* expression in the antibiotic producer as well as receiver. The regulation of *azoR* in all three THOR members and the koreenceine-dependent regulation of *clpP* in *F. johnsoniae* and B. cereus was validated by qRT-PCR (see Fig. S5).

10.1128/mBio.02486-21.8 FIG S5 Validation of THOR RNA-Seq gene expression changes using qRT-PCR. The fold change (FC) in genes was determined by qRT-PCR (white columns) on cDNA from the indicated samples matches trends seen in the RNA-Seq analysis (grey columns). (A) *P. koreensis azoR* was downregulated in the Δ*kec* samples. *F. johnsoniae* (B) *azoR* and (C) *clpP* were upregulated in the presence of *P. koreensis* but not regulated in the presence of Δ*kec.* B. cereus *azoR* (D), *clpP* (E), and *fsa* (F) were upregulated only in the presence of wild-type *P. koreensis* and not in the presence of Δ*kec.* Furthermore, slight reductions in fold change in the community compared to the pairwise seen in RNA-Seq (C, D, and F) were also seen by qRT-PCR. (G) Correlation between RNA-Seq and qRT-PCR fold change in genes from panels A to F showed strong alignment (*R*^2^ = 0.9273). Species-specific primers to the single copy *gyrA* in each member of THOR was used as a reference gene to normalize qRT-PCR results. ND, not determined. Download FIG S5, TIF file, 52.2 MB. Copyright © 2022 Hurley et al. 2022 Hurley et al. https://creativecommons.org/licenses/by/4.0/ This content is distributed under the terms of the Creative Commons Attribution 4.0 International license.

A minority of differentially expressed genes in response to koreenceine were shared between *F. johnsoniae* and B. cereus; the majority of functional enrichments were unique (Fig. 6). *F. johnsoniae* exhibited an enrichment for genes involved in lipid metabolism (Fig. 6A, COG I) in both upregulated and downregulated subsets, suggesting a targeted remodeling of the membrane (40, 41) or altered metabolism in general due to the reduced growth of *F. johnsoniae* with wild type *P. koreensis* not seen in B. cereus. *F. johnsoniae* also displayed a strong enrichment for downregulated genes related to extracellular structures (Fig. 6A, COG W). Both upregulated and downregulated genes in the functional category of inorganic ion transport and metabolism (Fig. 6B, COG P) were enriched in B. cereus, suggesting a specific intracellular ion enrichment. Also, exclusively in B. cereus, the COG functional categories of transcription and amino acid metabolism and transport (Fig. 6B, COG K and E, respectively) were upregulated. Two COG functional categories were enriched in opposite directions between the two THOR members—energy production and conversion (COG C) was upregulated in B. cereus and downregulated in *F. johnsoniae* in response to koreenceine. Genes involved in secondary metabolism genes (COG Q) displayed the opposite pattern; they were enriched among upregulated genes in *F. johnsoniae* and downregulated genes in B. cereus.

### Reduced *P. koreensis* inoculum enhances expression of unannotated genes.

**FIG 7:**
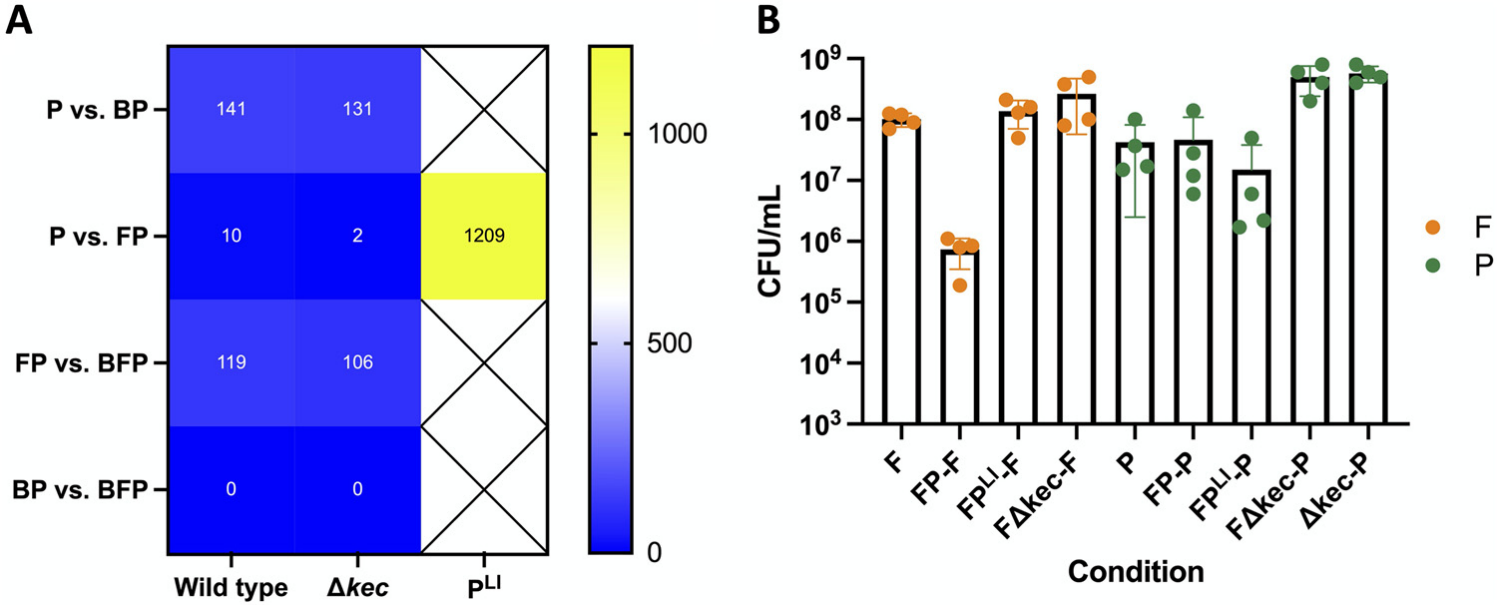
Relative abundance of *P. koreensis*, not presence of koreenceine, drives changes in *P. koreensis* gene expression. (A) Heat map showing number of *P. koreensis* DEGs (>2-fold) in response to category I and category II comparisons for 1 × 10^6^ wild-type, 1 × 10^6^ Δ*kec* mutant, or 2 × 10^4^ wild-type (P^LI^) CFU/mL *P. koreensis* inoculations. (B) Cell density (CFU/mL) of indicated conditions at the time of RNA harvest with *F. johnsoniae* levels shown in orange and *P. koreensis* levels shown in green.

Production of the antibiotic koreenceine mediated much of the pairwise *P. koreensis* expression changes in B. cereus and *F. johnsoniae*. We next sought to determine whether loss of antibiotic production in *P. koreensis* could enhance a cognate transcriptional response, reasoning the antibiotic-producing, most abundant member would not need to “listen” and adapt to its coculture partners. While the Δ*kec* mutant increased *F. johnsoniae* populations, which could have initiated increased interspecies signaling, the Δ*kec* mutant was not more sensitive to the presence of microbial partners than the wild type. In fact, the number of *P. koreensis* DEGs (>2-fold) in coculture with other THOR members was slightly reduced in the presence of the Δ*kec* mutant compared with the wild type (Fig. 7A).

The Δ*kec* mutant displayed a small growth advantage compared to wild type (Fig. 7B) so that even though *F. johnsoniae* growth was enhanced in the absence of koreenceine, *P. koreensis* was still the most abundant member of the community. To determine whether relative abundance could enhance *P. koreensis* response to *F. johnsoniae*, we tested the effect of reducing the inoculum of *P. koreensis* (P^LI^). Indeed, when its inoculum was reduced 50-fold, *P. koreensis* achieved the same population size as in the higher inoculum condition, but *F. johnsoniae* growth was unimpaired (Fig. 7B). The koreenceine biosynthesis genes (*kecA* to *kecK*) were downregulated an average of 12.5-fold in the P^LI^ condition compared to wild type alone, suggesting that the lower levels of koreenceine enabled *F. johnsoniae* growth, and that koreenceine genes were transcriptionally regulated in the presence of *F. johnsoniae.* Whereas it was largely unresponsive to *F. johnsoniae* under the equal inoculum conditions, when *P. koreensis* was initially at a numerical disadvantage, differential gene regulation increased >100-fold in response to *F. johnsoniae* (Fig. 7A).

**FIG 8:**
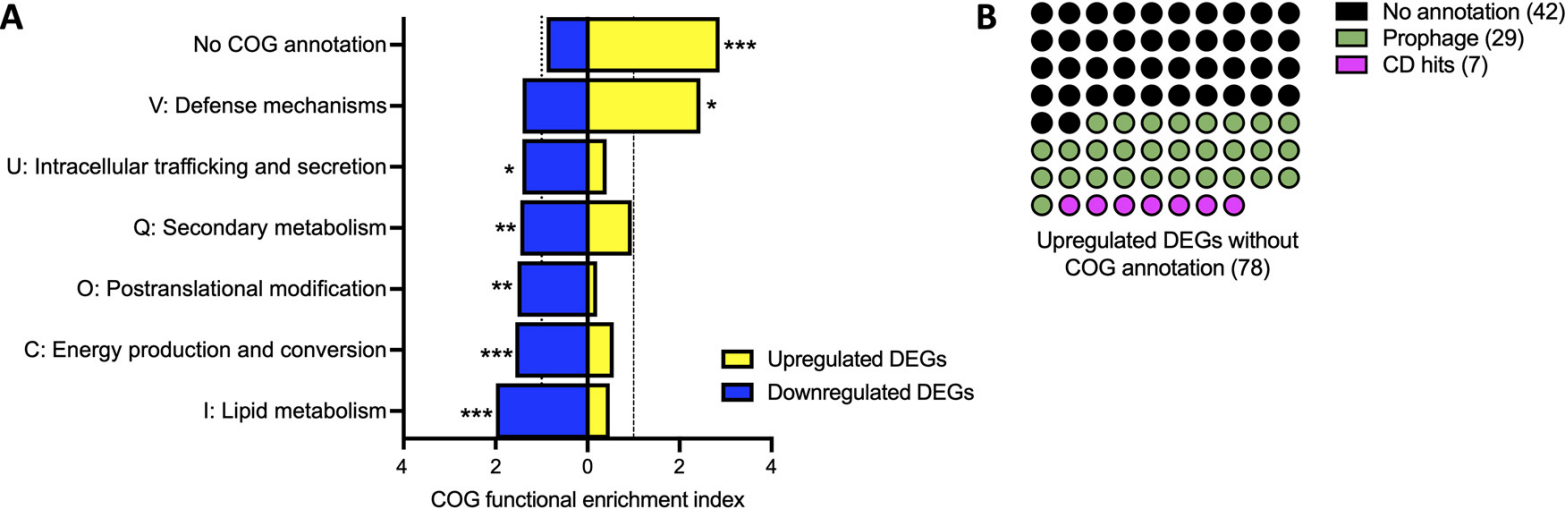
The major category of *P. koreensis* genes upregulated in response to *F. johnsoniae* have no functional annotation. (A) *P. koreensis* genome was functionally annotated using eggNOG and the baseline prevalence of each COG category was quantified. *P. koreensis* low inoculum genes sets were identified as >2-fold change in coculture with *F. johnsoniae* compared to *P. koreensis* alone. COG functional enrichment index was quantified as the percentage of COG category present in the FP^LI^ data set divided by the percentage of that particular COG category in the whole genome. To be enriched, the functional annotation must have an index greater than 1 (dotted lines), meaning the frequency of that function in the data set is greater than found in the genome. Upregulated genes shown in yellow and downregulated genes shown in blue. Only COG categories with significant enrichment are shown. Significance was determined in Rstudio by Fisher exact test (*, *P < *0.05; **, *P < *0.01; ***, *P < *0.001). (B) Upregulated DEGs without COG annotation were searched for conserved domains (CD) and the frequency of CD hits, prophage genes, and remaining unannotated genes are shown.

Next, we determined which functional categories were enriched in the *P. koreensis* DEGs in response to *F. johnsoniae* under the low-inoculum condition compared to *P. koreensis* alone, which achieved similar populations at harvest even with different inoculum sizes (Fig. 7B). The koreenceine biosynthetic gene cluster was downregulated, so we hypothesized a general reduction in secondary metabolism. As predicted, secondary metabolism (COG Q) genes were enriched among the downregulated DEG subset (Fig. 8A). Genes without a COG annotation exhibited the greatest functional enrichment of either up- or downregulated DEGs under the low *P. koreensis* inoculum in response to *F. johnsoniae.* Further exploration into nearby genes in the *P. koreensis* genome revealed that 29 of the 78 unannotated genes likely belong to two integrated phages. Of the remaining genes, seven could be annotated from the National Center for Biotechnology Information’s conserved domain database (42), whereas the other 42 genes remained unannotated (Fig. 8B). These data show that *P. koreensis* genes turned on by coculture with *F. johnsoniae* were enriched for unannotated genes, consistent with our prediction that many genes involved specifically in community responses have not been characterized in pure culture.

## DISCUSSION

Transcriptionally profiling the model microbiome THOR revealed unique communication strategies among its members. In pairwise coculture, expression of hundreds of *F. johnsoniae* genes changed in response to either *P. koreensis* or B. cereus. Similarly, B. cereus responded to coculture with *P. koreensis*, but expression of only a handful of genes changed in response to *F. johnsoniae* despite the demonstrated interactions of hitchhiking and peptidoglycan cross-feeding (28) between the two species. The lack of clear evidence from the transcript analysis for the mechanisms underpinning those interactions suggests they require additional environmental signals or are not transcriptionally regulated. *P. koreensis*, in contrast, when inoculated at similar populations as the other THOR members, became the most abundant species, inducing massive gene expression changes in its coculture partners while changing very little of its own expression profile (Fig. 1 and 2).

*P. koreensis* gene expression was substantially affected by coculture only when *P. koreensis* was placed at a numerical disadvantage in the community (Fig. 7A), suggesting that as a minority member of the community *P. koreensis* needs to sense its surroundings more precisely. Comparing the low-inoculum and Δ*kec* conditions, both exhibited reduced koreenceine expression and increased *F. johnsoniae* abundance, but only P^LI^ increased *P. koreensis* gene regulation. Thus, population ratios and koreenceine expression seem to be interconnected and koreenceine has temporal importance. As a minority community member, *P. koreensis* may need to “listen” more to the members in the majority, perhaps to take advantage of nutritional opportunities and protect against antagonism (43, 44). Exploring this phenomenon in microbial communities from disparate environments will determine its applicability in microbial systems under different conditions or with different community composition.

In addition to inducing the most gene expression changes in THOR, *P. koreensis* pairwise interactions were largely maintained in the full three-species community setting (Fig. 3C and D; see also Fig. 4A and B), even if the interaction was an absence of regulation (Fig. 4D). The dominance of *P. koreensis* in THOR largely stemmed from the production of the antibiotic koreenceine (22), which we refer to as THOR’s hammer because of its powerful impact. In pairwise coculture with *P. koreensis*, koreenceine was responsible for 85% of signaling in *F. johnsoniae* and 22% of signaling in B. cereus, although B. cereus growth was not impaired, unlike *F. johnsoniae*. There was a shared response to koreenceine, mainly upregulating stress response and downregulating translation genes. B. cereus turning on stress response implies koreenceine may negatively affect B. cereus growth, but B. cereus can either inactivate or mitigate the effects of koreenceine and survive the assault. The functional response to the Δ*kec* mutant was largely unique to each member. Other studies have shown both unique transcriptional response of different strains to the same antibiotics (45) or, more frequently, unique transcriptional response of a bacterial species to sublethal levels of different antibiotics (46, 47). Such work has explored the role of antibiotics as signaling molecules rather than as weapons (48–54). Here, the case may be both, especially for *F. johnsoniae*, whose growth is certainly inhibited but never fully eradicated by koreenceine. Similarly, in another soil coculture system, secondary metabolites can kill or activate quorum-sensing gene products, depending on their concentration (55). Further studies exploring metabolomic shifts of THOR with and without the Δ*kec* mutant may provide insight into the functional response to an endogenously produced antibiotic within a microbiome, linking molecules to the demonstrated transcriptional changes.

Unique microbial coculture conditions, even simply modifying inoculum ratios, could be a powerful tool for discovering new gene functions. Almost one quarter of *P. koreensis* genes upregulated in the low-inoculum condition lacked both COG and NCBI conserved domain annotations. Further inquiry with biochemical and genetic tools will reveal the functions of these genes. Upon exploring the genes lacking COG annotation, we found many belonged to predicted prophage genomes (29/78 genes). Transcriptional activation of prophage genes in response to interspecies coculture has been observed before, most intriguingly as a mechanism by which rare community members gain a competitive advantage against more abundant, susceptible neighbors (56–58). Whether phage particles are produced by *P. koreensis* in coculture remains to be determined.

Comparative metatranscriptomics of the model microbiome, THOR, revealed intricate patterns of interspecies interactions. One expected pattern for community-specific gene expression would be genes that are only regulated in the presence of all three THOR members. Indeed, we found 12 genes in *F. johnsoniae* and 11 genes in B. cereus with no pairwise interactions (category I) that exhibited regulation in category II and at least >2-fold regulation in the community (category III). Although we detected this pattern of expression, it was not a major trend in the data set. We found the community-specific gene expression patterns were more subtle: genes were often regulated by one partner in pairwise culture and both partners in the community, which we define as “higher-order” regulation. Such gene regulation is an emergent property of the community. Based on the pairwise data, we would not predict the neutral partner in the pairwise condition to affect those genes in the community. For example, *F. johnsoniae* had more influence on B. cereus gene expression in the presence of *P. koreensis* than in its absence. Overall, gene expression in the THOR community neither phenocopied the dominant pairwise interactions nor was the additive result of both pairwise conditions (Fig. 3 and 4), suggesting the intracellular molecular environment of the species within the community is unique. In particular, when *P. koreensis* was at a numerical disadvantage, it responded to coculture by altering expression of more than 1,000 genes. Of great interest is the finding that upregulated genes are enriched for genes of unknown function. This is consistent with the prediction that if bacteria contain genes dedicated only to life in a community, then it is likely that the functions of those genes would be largely unstudied to date since most functional gene assignments have been made in studies of pure cultures.

The *P. koreensis* antibiotic koreenceine appears to serve as THOR’s hammer, responsible for global shifts in transcriptional response in coculture partners. This single molecule drives the expression of thousands of genes and globally affects the community interaction networks. The mechanism of global regulation by koreenceine and the functions of the many genes of unknown function identified in this study will be fruitful avenues for further investigation. This study demonstrates the cascade of interspecies interactions that respond to perturbation, even in a simple community. It remains to be determined whether the same principles governing behavior in this model apply to more complex microbiomes.

## MATERIALS AND METHODS

### Bacterial strains and culture conditions.

Bacillus cereus UW85 (B), Flavobacterium johnsoniae UW101 (F), Pseudomonas koreensis CI12 (P), and Pseudomonas koreensis CI12 Δ*kecA-K*::*tetRA* mutant (22) were propagated on Luria-Bertani (LB) agar at 28°C and grown overnight in liquid culture in half-strength tryptic soy broth (TSB) at 28°C with vigorous shaking.

### Community abundance assay.

Strains were grown individually for 20 h at 28°C with vigorous shaking. One-milliliter samples from each overnight culture were removed, the cells were washed once and resuspended in 10 mM NaCl, and the cell density was determined spectrophotometrically at an optical density at 600 nm (OD_600_). The OD_600_ corresponding to 10^6^ cells mL^−1^ for each species was determined in a previous experiment. Cultures (1/10-strength TSB) were inoculated with 1 × 10^6^ *F. johnsoniae* cells mL^−1^ (final OD_600_ = 0.0008), 1 × 10^6^ B. cereus cells mL^−1^ (final OD_600_ = 0.0167), and/or 1 × 10^6^ *P. koreensis* cells mL^−1^ (final OD_600_ = 0.016) alone, pairwise (2 × 10^6^ total bacterial cells mL^−1^), or in the complete community (3 × 10^6^ total bacterial cells mL^−1^). For the low-inoculum condition (FP^LI^), *F. johnsoniae* was inoculated at 1 × 10^6^ cells mL^−1^ and *P. koreensis* was inoculated at 2 × 10^4^ cells mL^−1^. One-milliliter aliquots were dispensed into 14-mL glass culture tubes and incubated statically at 20°C in the dark to simulate conditions experienced by the bacteria in their soil or root habitats. At each time point, individual culture tubes were removed for destructive sampling. Tubes were vortexed for 30 s, sonicated in a water bath for 2 min, and vortexed again for 30 s before subjecting the cultures to a 1:10 dilution in 10 mM NaCl in a 96-well plate. Aliquots (5 μL) were dispensed on species-specific media in technical duplicate to determine cell density by dilution plating. LB medium with polymyxin B (5 mg mL^−1^) and gentamicin (10 mg mL^−1^) selected for B. cereus and *F. johnsoniae*, respectively, and a combination of ampicillin (100 mg mL^−1^) and erythromycin (5 mg mL^−1^) selected for *P. koreensis.* Plates were incubated at 28°C for 1 day for B. cereus and *P. koreensis* and 2 days for *F. johnsoniae*, and then the colonies were counted.

### RNA harvest.

Five culture tubes of the same condition after 19.5 h at 20°C were pooled (5 mL total) and added to 10 mL of RNAprotect (Qiagen, catalog no. 76526), vortexed, and incubated for 15 min at room temperature. The cells were pelleted and washed once with 10 mM NaCl and then lysed mechanically by freezing the pellets in liquid nitrogen and grinding them with a sterile pestle. Filtered pipette tips were used for the rest of the experiment to protect RNA. Phosphate-buffered saline (200 μL) was used to rinse the pestle and resuspend the cell lysate. TRIzol (Fisher Scientific, catalog no. 15596026) was heated to 65°C, and 1 mL was added to each lysate. Samples were then heated at 65°C for 2 min, frozen at −80°C for 20 min, and thawed at room temperature. Samples were transferred to 2-mL microcentrifuge tubes containing 240 μL of chloroform, inverted 20 times to mix them, and incubated at room temperature for 3 min. Samples were centrifuged at 4°C for 30 min at 12,000 rpm, 600 μL of the aqueous layer was added to 600 μL of cold isopropanol, and the tubes were inverted 20 times and incubated at room temperature for 10 min. Pellets of the centrifuged samples were dried in a biosafety cabinet for 10 to 15 min and resuspended in RNase-free water. A maximum of 20 mg of RNA was digested with Turbo DNase (Thermo Fisher, catalog no. AM2238) at 37°C for 30 min and cleaned up with phase-lock tubes. The aqueous layer was subjected to ethanol precipitation. Pellets were washed twice with 70% ethanol, dried, and resuspended in RNase-free water.

### RNA sequencing.

DNase-treated RNA samples were submitted to the University of Wisconsin Biotechnology Center for RNA sequencing. In brief, samples were run on an Agilent Bioanalyzer for quality control. 500 ng of each sample was depleted of rRNA with RiboZero Plus before cDNA synthesis with the TruSeq Stranded Total RNA Library Prep and sequenced on an Illumina NovaSeq 6000. The first data set sequenced included seven samples (B, F, P, BF, BP, FP, and BFP) in biological quadruplicate, and the second data set sequenced included nine samples (P, BP, FP, BFP, Δ*kec*, BΔ*kec*, FΔ*kec*, BFΔ*kec*, and FP^LI^) in biological quadruplicate. All samples were sequenced at a depth of 5 million reads, except for conditions in which *F. johnsoniae* was inhibited by the presence of *P. koreensis.* These samples (FP and BFP) were sequenced to 50 million reads to capture enough reads from the low-abundance member, *F. johnsoniae.* For the Δ*kec* sequencing data set, FP and BFP samples were sequenced to a depth of 20 million reads.

### RNA-Seq data analysis.

Samples were submitted to FastQC by the University of Wisconsin Biotechnology Center for quality control and to remove sequencing adapters. Forward and reverse reads were paired by Fastp (59). Reads were then competitively separated against all three THOR genomes with BBSplit (https://sourceforge.net/projects/bbmap/), tossing ambiguously mapped reads. The interleaved .fq files were mapped to individually indexed genomes with Bowtie 2 (60). Mapped reads per gene were quantified with HTSeq (61) on -intersection-strict against genomes annotated by Prokka (62). Comparing the total reads after Fastp analysis to the number of reads in the species-specific .fq files after bbsplit, we find that, on average, 0.015-0.702% of reads for each condition were either unmapped or tossed because of ambiguity. Reads that mapped to the genomes of species not present in the sample was even more rare: 0.00002 to 0.0016% on average. The overwhelming majority of reads in the samples mapped to genomes of species present (ranging from 99.3% in BF to >99.9% in B, F, or P alone) as seen in Data Set S1.

10.1128/mBio.02486-21.1 DATA SET S1 Species reads. Total reads, mapped reads, and tossed/unmapped reads are shown for each species under all conditions. Download Data Set S1, XLSX file, 0.02 MB. Copyright © 2022 Hurley et al. 2022 Hurley et al. https://creativecommons.org/licenses/by/4.0/ This content is distributed under the terms of the Creative Commons Attribution 4.0 International license.

To reduce noise caused by genes with very low expression levels in the data set, only genes with at least 10 reads across all conditions (for example, F, BF, FP, and BFP) were used to identify differentially expressed genes (DEGs) with an FDR adjusted *P* value of <0.05 in edgeR (63). Total lists of significant DEGs with log_2_-fold change for each species can be found in Data Set S2. Volcano plots were made with ggplot2 (64) in R.

10.1128/mBio.02486-21.2 DATA SET S2 DEGs. The gene IDs, functions, and log_2_-fold changes for DEGs with FDR *P* value <0.05 are shown for category I, II, and III comparisons for B. cereus (tab 1), *F. johnsoniae* (tab 2), *P. koreensis* (tab 3), B. cereus with Δ*kec* (tab 4), *F. johnsoniae* with Δ*kec* (tab 5), and Δ*kec* (tab 6). Download Data Set S2, XLSX file, 1.3 MB. Copyright © 2022 Hurley et al. 2022 Hurley et al. https://creativecommons.org/licenses/by/4.0/ This content is distributed under the terms of the Creative Commons Attribution 4.0 International license.

For the expression level of single-species pairwise genes in other coculture conditions in Fig. 3 and 4 (see also Fig. S3), the normalized counts per million (cpm) from edgeR for each DEG was averaged for each of the conditions. Then, the averaged values in the pairwise and BFP conditions were each divided by the average in the species alone for each DEG to determine the change in expression level in coculture conditions compared to the pure culture. For the calculated sum of both pairwise interactions, the following formula (65) using the averaged cpm from PC (“pure culture” of the species) and the two pairwise coculture (A and B) was applied: Sum = 10^(logA – logPC) + (logB – logPC)^. Because of the non-normal distributions and wide range of values, the expression levels and the calculated sum were then converted to log. Results were visualized with GraphPad Prism version 9.2.0 for macOS and statistics were determined using the exactRankTests package (https://cran.r-project.org/web/packages/exactRankTests/index.html) in RStudio, specifically the wilcox.exact (66) and then the p.adjust with “bonferroni” methods.

### COG functional enrichments.

Each THOR genome was annotated using eggNOG-mapper v2 (67; http://eggnog-mapper.embl.de/). The baseline frequency of COG categories was then calculated for each genome. For Fig. 6, genes that were upregulated in pairwise coculture with *P. koreensis* that were downregulated in comparison to the pairwise with Δ*kec* were considered koreenceine-specific upregulated genes. Similarly, the downregulated koreenceine-specific subset of genes required an upregulation in comparison to the pairwise with Δ*kec.* There was a >2-fold cutoff for fold change with wild-type *P. koreensis* but no fold change cut off for the Δ*kec* comparison. The frequency of COG categories in the upregulated and downregulated koreenceine-specific gene subsets for B. cereus and *F. johnsoniae* was determined and compared to the frequency in the whole genome. A Fisher exact test with Bonferroni correction in RStudio was used to determine the significance of COG category enrichments. The same pipeline was applied to genes regulated >2-fold in *P. koreensis* in the low-inoculum condition with *F. johnsoniae* (FP^LI^).

### Species-specific qRT-PCR primer design and sequencing.

The single copy *gyrA* gene was selected as the housekeeping reference gene for qRT-PCR (the copy number of the 16S rRNA gene differs among the THOR members). The *gyrA* sequences from each species were aligned and variable regions were selected for species-specific primer design. Similarly, primers for the genes in Fig. S1 were designed using the NCBI Primer-BLAST tool (68) to be specific to only the specified gene within the designated species. Primers were validated to not amplify regions of noncognate species (see Fig. S6A to C). cDNA was made from the RNA extraction submitted to RNA-Seq with SuperScript III first strand synthesis system (Thermo Fisher, catalog no. 18080051).

10.1128/mBio.02486-21.9 FIG S6 Species specificity of *gyrA* primers and products of qRT-PCR. Each *gyrA* primer set from Table S1 was tested against 5 ng of cDNA from each THOR member. (A to C) Only B. cereus (purple) cDNA amplifies with UW85gyrA primers (A), only *F. johnsoniae* (orange) cDNA amplifies with UW101gyrA primers (B), and only *P. koreensis* (green) cDNA amplifies with CI12gyrA primers (C). (D) qRT-PCR products from the primer sets amplifying their cognate pure species cDNA in Table S2 for the genes shown in panel E. Lanes 9, 10, 14, and 15 were not pursued in this study and will not be found in Table S1. Download FIG S6, TIF file, 41.3 MB. Copyright © 2022 Hurley et al. 2022 Hurley et al. https://creativecommons.org/licenses/by/4.0/ This content is distributed under the terms of the Creative Commons Attribution 4.0 International license.

cDNA titrations with *gyrA* primers determined that 5 ng of template was within the dynamic range of all three species. Thus, 5 ng of template was amplified in technical quadruplicate and biological triplicate using the primers in Table S1 with PowerUp SYBR green Master Mix (Thermo Fisher, catalog no. A25742) on a Bio-Rad CFX96 real-time system (see Fig. S6D and E). Outliers in the technical quadruplicates were determined by the Grubb’s test and removed from the analysis. Fold change was calculated using the 2^–ΔΔ^*^CT^* method in Excel with species-specific *gyrA* as the reference gene and the wild-type pure culture as a control. Results were visualized with GraphPad Prism version 9.2.0 for macOS.

10.1128/mBio.02486-21.10 TABLE S1 Primers used for qRT-PCR in this study. The species specificity of *gyrA* primers and gel validation of qRT-PCR products can be found in Fig. S6. Download Table S1, TIF file, 41.3 MB. Copyright © 2022 Hurley et al. 2022 Hurley et al. https://creativecommons.org/licenses/by/4.0/ This content is distributed under the terms of the Creative Commons Attribution 4.0 International license.
