# Supplementary figures and images for: THOR’s Hammer: the Antibiotic Koreenceine Drives Gene Expression in a Model Microbial Community
Source: mBio. 2022 Apr 18;13(3):e02486-21. doi: 10.1128/mbio.02486-21 (PMC9239112; doi:10.1128/mbio.02486-21)

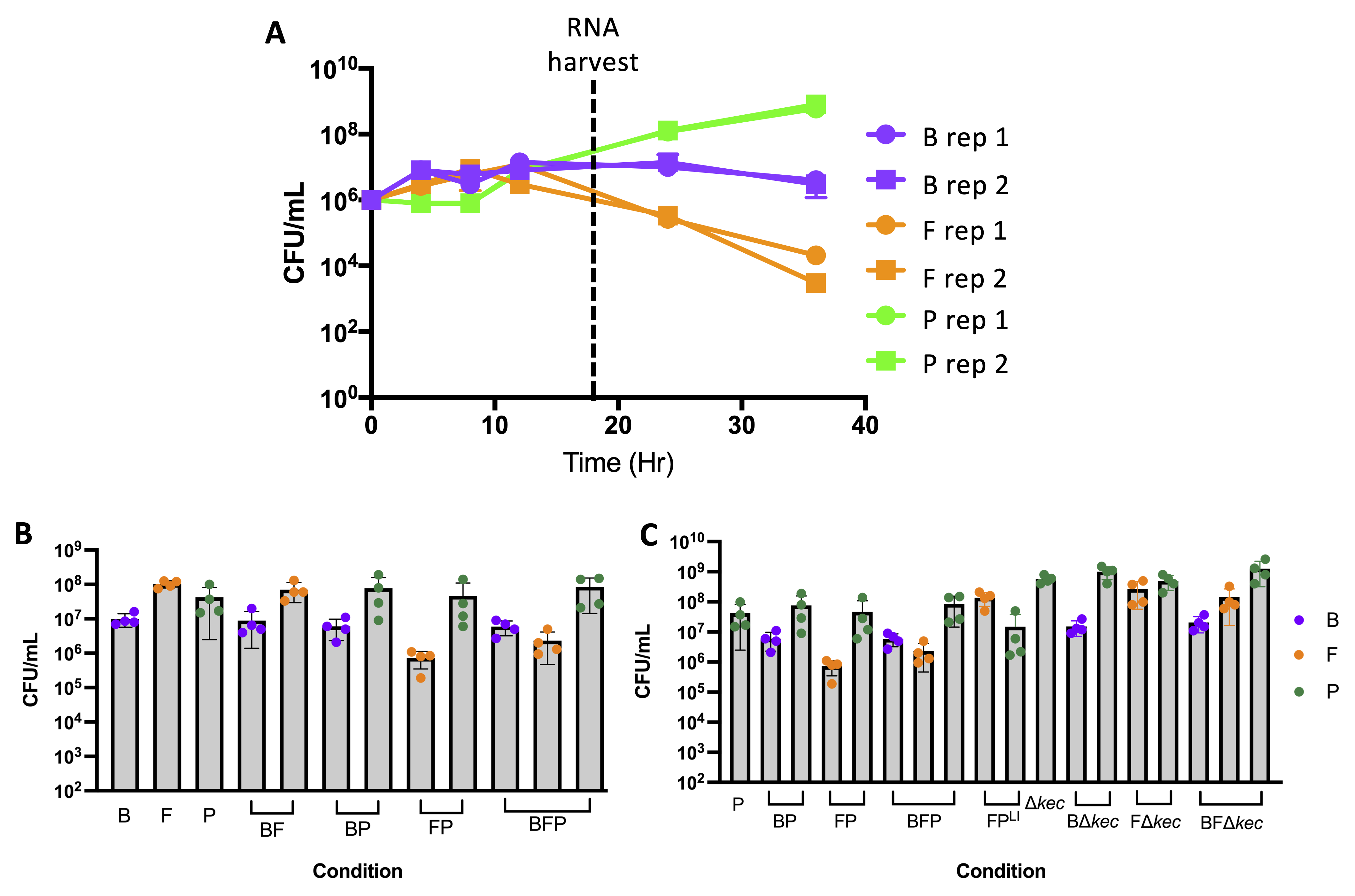

Supplement: FIG S1 [file mbio.02486-21-s0001.tif]

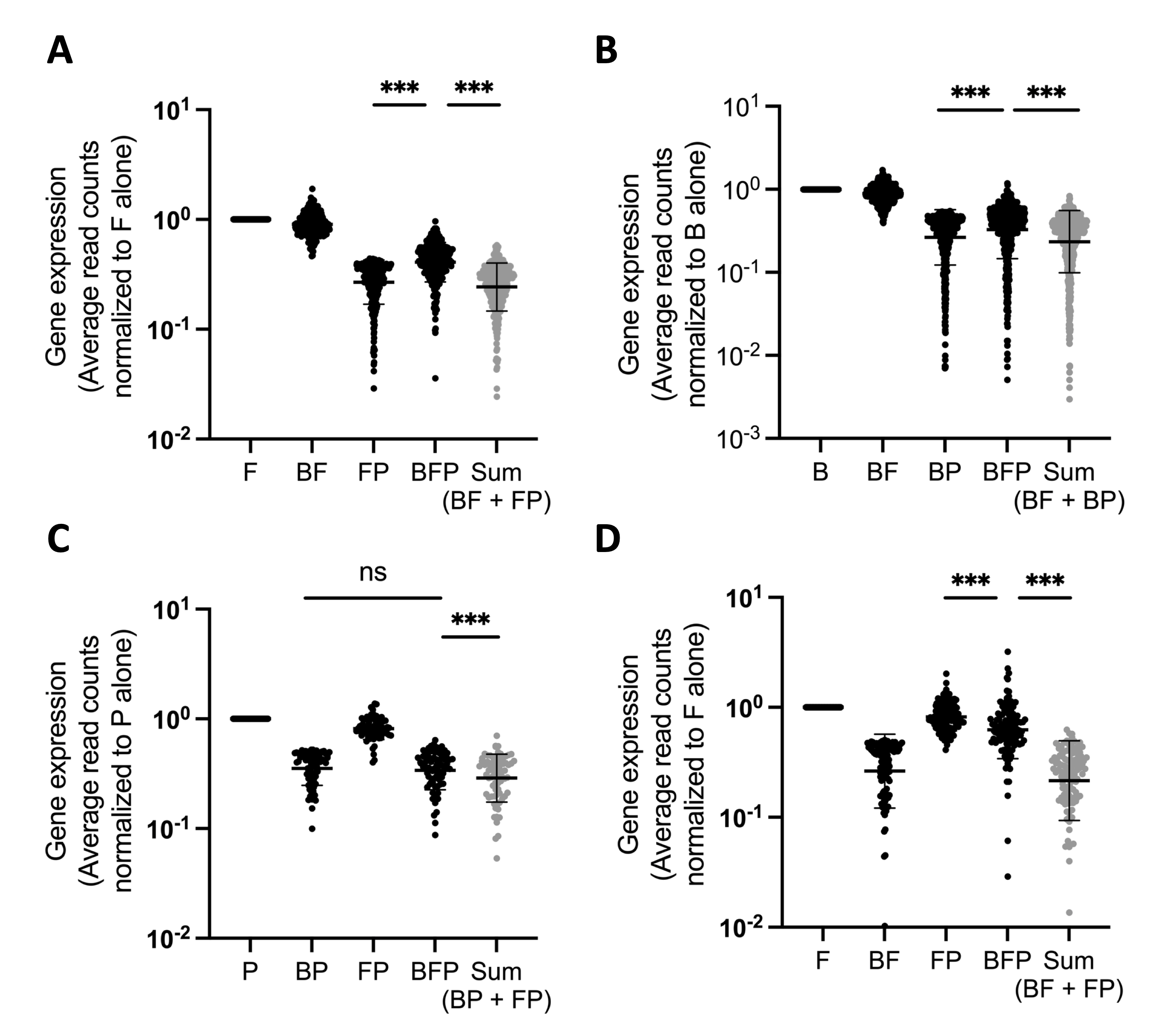

Supplement: FIG S3 [file mbio.02486-21-s0003.tiff]

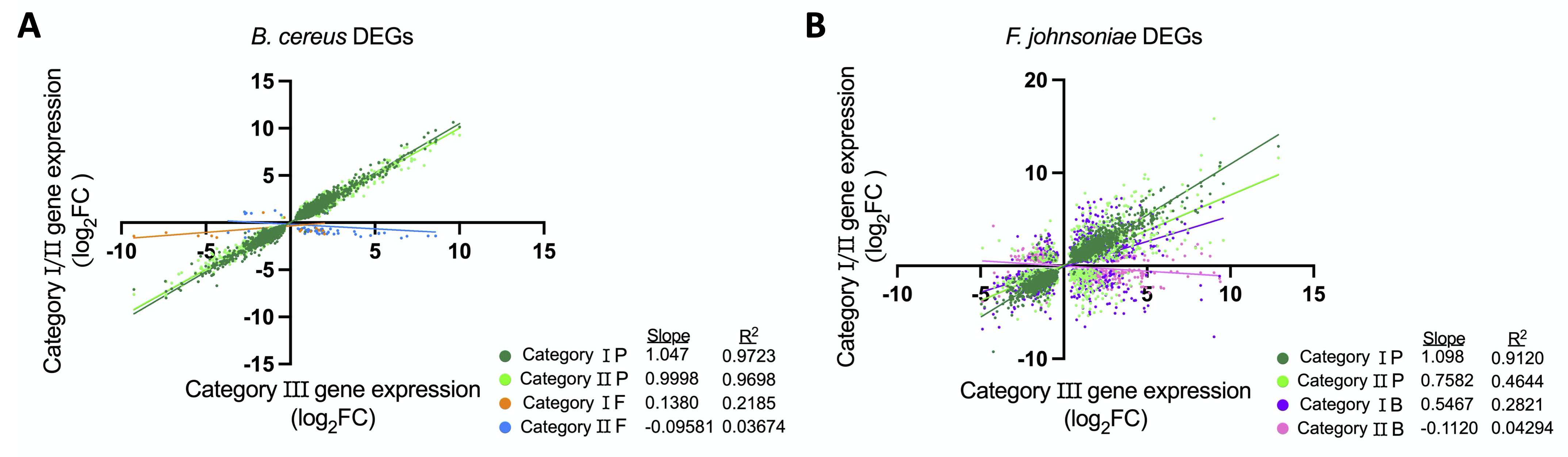

Supplement: FIG S4 [file mbio.02486-21-s0004.tif]

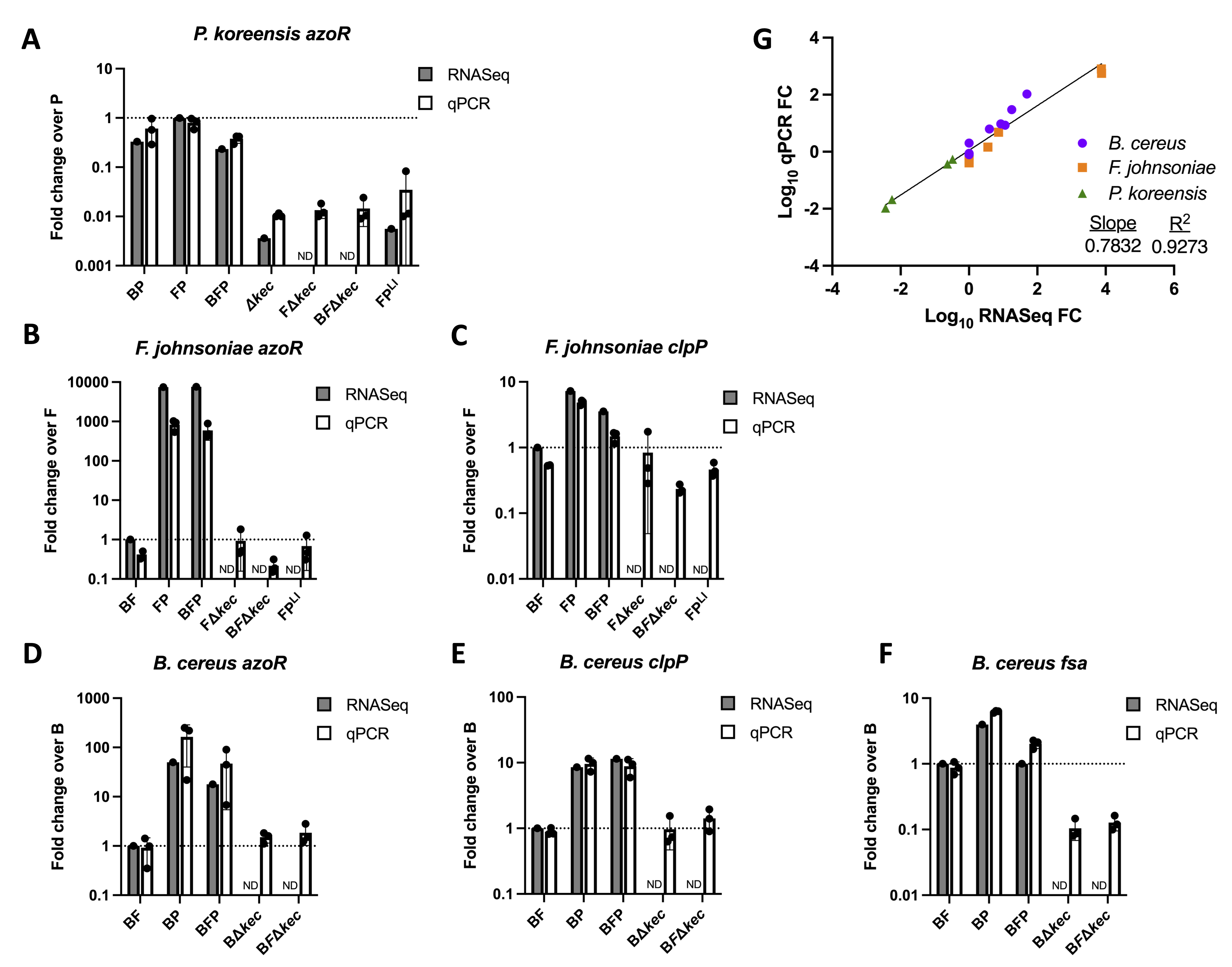

Supplement: FIG S5 [file mbio.02486-21-s0005.tif]

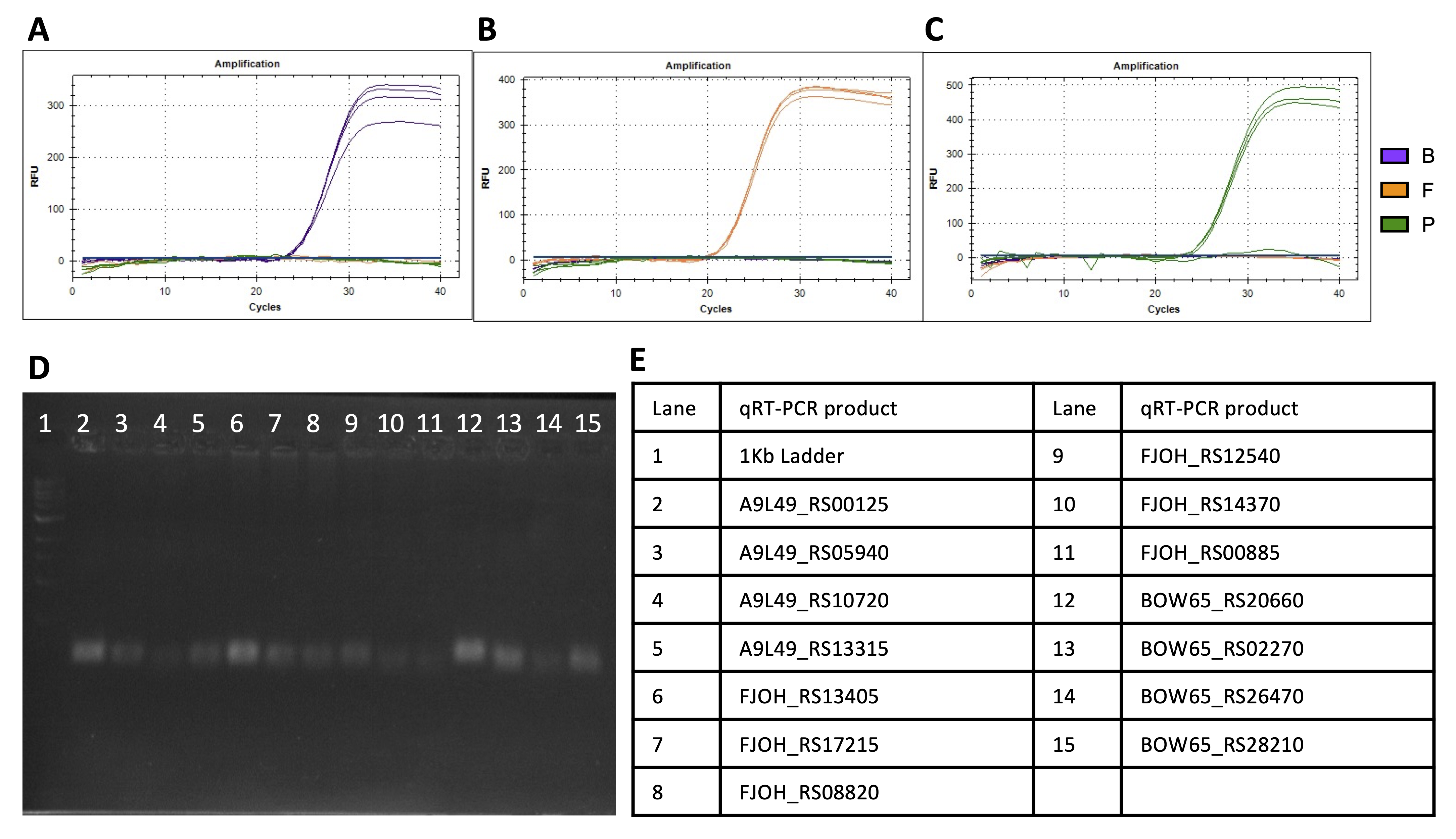

Supplement: FIG S6 [file mbio.02486-21-s0006.tif]

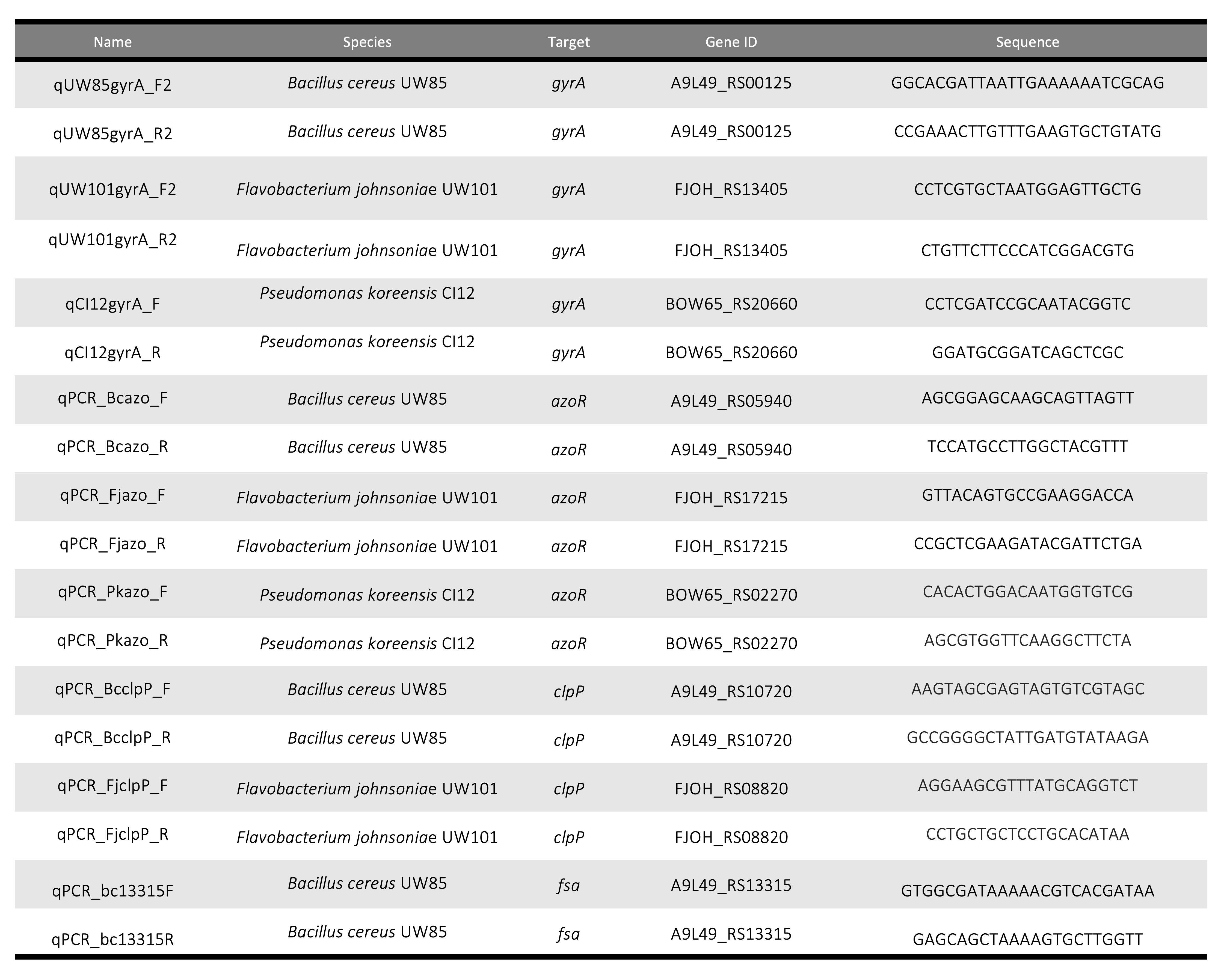

Supplement: TABLE S1 [file mbio.02486-21-s0007.tif]
